# Noise in Neurons and Synapses Enables Reliable Associative Memory Storage in Local Cortical Circuits

**DOI:** 10.1523/ENEURO.0302-20.2020

**Published:** 2021-02-23

**Authors:** Chi Zhang, Danke Zhang, Armen Stepanyants

**Affiliations:** 1 Department of Physics and Center for Interdisciplinary Research on Complex Systems, Northeastern University, Boston, MA 02115; 2 CAS Key Laboratory of Brain Connectome and Manipulation, Interdisciplinary Center for Brain Information, The Brain Cognition and Brain Disease Institute, Shenzhen Institutes of Advanced Technology, Chinese Academy of Sciences, Shenzhen-Hong Kong Institute of Brain Science-Shenzhen Fundamental Research Institutions, Shenzhen, Guangdong China

**Keywords:** associative learning, memory retrieval, perceptron, replica, spiking errors, synaptic noise

## Abstract

Neural networks in the brain can function reliably despite various sources of errors and noise present at every step of signal transmission. These sources include errors in the presynaptic inputs to the neurons, noise in synaptic transmission, and fluctuations in the neurons’ postsynaptic potentials (PSPs). Collectively they lead to errors in the neurons’ outputs which are, in turn, injected into the network. Does unreliable network activity hinder fundamental functions of the brain, such as learning and memory retrieval? To explore this question, this article examines the effects of errors and noise on the properties of model networks of inhibitory and excitatory neurons involved in associative sequence learning. The associative learning problem is solved analytically and numerically, and it is also shown how memory sequences can be loaded into the network with a biologically more plausible perceptron-type learning rule. Interestingly, the results reveal that errors and noise during learning increase the probability of memory recall. There is a trade-off between the capacity and reliability of stored memories, and, noise during learning is required for optimal retrieval of stored information. What is more, networks loaded with associative memories to capacity display many structural and dynamical features observed in local cortical circuits in mammals. Based on the similarities between the associative and cortical networks, this article predicts that connections originating from more unreliable neurons or neuron classes in the cortex are more likely to be depressed or eliminated during learning, while connections onto noisier neurons or neuron classes have lower probabilities and higher weights.

## Significance Statement

Signal transmission in the brain is accompanied by many sources of errors and noise, and yet, neural networks can reliably store memories. This article argues that noise should not be viewed as a nuisance, but that it is an essential component of the reliable learning mechanism implemented by the brain. The article describes a network model of associative sequence learning, showing that for optimal retrieval of stored information learning must be conducted in the presence of noise. To validate the model, it is shown that associative memories can be loaded into the network with an online perceptron-type learning rule and that networks loaded to capacity develop many structural and dynamical properties observed in the brain.

## Introduction

Brain networks can reliably store and retrieve long-term memories despite the facts that various sources of errors and noise accompany every step of signal transmission through the network (Faisal et al., 2008), synaptic connectivity changes over time (Trachtenberg et al., 2002; Holtmaat and Svoboda, 2009; Gala et al., 2017), and extraneous sensory inputs are usually present during memory recall. The brain can reduce the effects of noise and extraneous inputs by attending to the memory retrieval process (Cohen and Maunsell, 2009; Mitchell et al., 2009), but such hindrances cannot be eliminated entirely. Therefore, the reliability required for memory retrieval must be built into the network during learning. This proposal presents an interesting challenge. Traditional supervised learning models, such as the ones that rely on the perceptron rule (Minsky and Papert, 1969; Hertz et al., 1991), modify connectivity only when a neuron’s output deviates from its target output. Thus, in such models learning stops as soon as the neuron produces the desired response and, subsequently, there is no possibility for improving the response reliability. The network connection weights in such models may end up near the boundary of the solution region, and a small amount of noise during memory retrieval can lead to errors or completely disrupt the retrieval process. More reliable solutions are located farther away from the solution region boundary, but the perceptron rule is not guaranteed to find them. Thus, it is not clear how the neural networks in the brain manage not only to learn but also to do it reliably.

**Figure 1.**
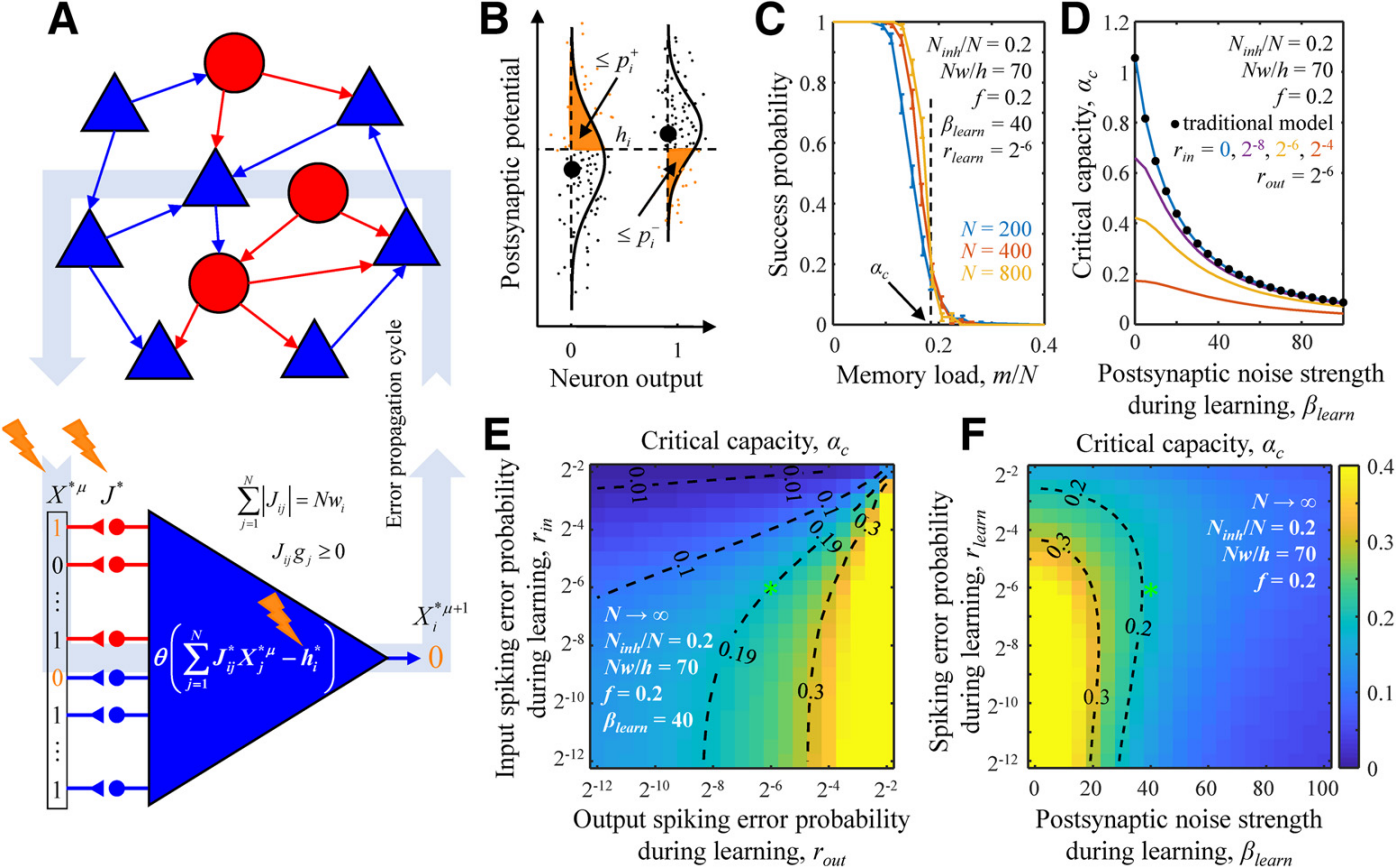
Associative memory storage in a recurrent network of inhibitory and excitatory neurons in the presence of errors and noise. ***A***, Error propagation through the network. Inhibitory neurons (red circles) and excitatory neurons (blue triangles) form an all-to-all potentially (structurally) connected network. Red and blue arrows represent actual (functional) connections. Spiking errors (errors contained in $X^{*}$), synaptic noise ($J_{ij}^{*}$), and intrinsic noise ($h_{i}^{*}$) accompany signal transmission (orange lightning signs). Errors in the neurons’ outputs at a given time step become spiking errors in the next time step. ***B***, Fluctuations in PSPs for two associations with target neuron outputs 0 (left) and 1 (right). Large black dots denote PSPs in the absence of errors and noise. Small dots represent PSPs on different trials in the presence of errors and noise. Orange areas to the left of the PSP probability densities (solid lines) represent the probabilities of erroneous spikes (left) and spike failures (right). ***C***, The probability of successful learning by a neuron is a sharply decreasing function of memory load *m*/*N*. Solid curves represent the probabilities of successful learning obtained with nonlinear optimization (see Materials and Methods) for neurons receiving *N *=* *200, 400, and 800 homogeneous inputs. The numerical values of *β_learn_* and *r_in_* = *r_out_* ≡ *r_learn_* are provided in the figure. The values of all other parameters of the model were adapted from Chapeton et al. (2015). At 0.5 success probability, the neuron is said to be loaded to capacity, *α*. The dashed black line represents the theoretical (critical) capacity, *α_c_*, obtained with the replica method in the *N* → ∞ limit. ***D***, *α_c_* as a function of *β_learn_* for different input noise strengths (colored lines). In the case of *r_in_* = 0, solution of Equation 1 (blue line) coincides with the solution of the traditional model (Zhang et al., 2019b), which uses a generic robustness parameter (black dots). ***E***, Map of *α_c_* for a neuron receiving homogeneous input as a function of *r_in_* and *r_out_*. ***F***, Same as a function of *β_learn_* and *r_in_* = *r_out_* ≡ *r_learn_*. The maps in ***E***, ***F*** were obtained with the replica method (see Materials and Methods), and the green asterisks correspond to the values of parameters used in ***C***. Dashed isocontours are drawn as a guide to the eye.

In the case of associative memory storage, reliability can be incorporated into the perceptron learning rule by means of a generic robustness parameter (Brunel et al., 2004). This traditional description, however, is not biologically motivated and does not account for various types of errors and noise present during learning and memory retrieval (Fig. 1*A*). A more comprehensive account must include errors in the inputs to the neurons, combine them with fluctuations in the neurons’ presynaptic connection weights and intrinsic sources of noise, and produce spiking errors in the neurons’ outputs. The latter, injected back into the network, give rise to input errors in the next time step. The recurrence of errors presents a clear challenge for the retrieval of associative memory sequences considered in this study. If not corrected at every step of the retrieval process, errors in the network activity can amplify over time and lead to an irreversible deviation of the retrieved trajectory from the loaded sequence, i.e., a partially retrieved memory.

The premise of this article is that errors and noise are essential components of the reliable learning mechanism implemented in the brain. As different fluctuations accompany the presentation of the same learning example to a neuron on different trials, the neuron in effect never stops learning. Its connection weights move further away from the solution region boundary every time a progressively larger fluctuation is encountered. This process increases the reliability of the loaded memory which can later be retrieved in the presence of noise. Similar ideas have been successfully used in machine learning where an augmentation of training examples with noise (Bishop, 1995) and dropping out neurons and connections (Srivastava et al., 2014) during training have been shown to significantly reduce both overfitting and training time. And, there are many other examples in which noise is put to a constructive use to improve various functions of physical and neural systems (for review, see Gammaitoni et al., 1998; Stein et al., 2005; McDonnell and Abbott, 2009; McDonnell and Ward, 2011). Therefore, the hypothesis that errors and noise are exploited by the brain for reliable memory storage may not be entirely surprising. Still, this hypothesis requires careful quantitative evaluation and validation with experimental data, which is the focus of this study.

## Materials and Methods

### Network model of associative memory storage in the presence of errors and noise

We considered a model of associative sequence learning by a local (∼100 μm in size), all-to-all potentially (structurally) connected (Stepanyants and Chklovskii, 2005; Stepanyants et al., 2008) cortical network, albeit with no synaptic input originating from outside the circuit. The model network consisted of *N_inh_* inhibitory and (*N* − *N_inh_*) excitatory McCulloch and Pitts neurons (McCulloch and Pitts, 1943; Fig. 1*A*) and was faced with a task of learning a sequence of consecutive network states, $X^{1}\to X^{2}\to ...X^{m+1}$, in which $X^{\mu }$ is a binary vector representing target activities of all neurons at a time step *μ*, and the ratio *m*/*N* is referred to as the memory load. Some assumptions and approximations of the model are discussed in (Chapeton et al., 2012). During learning, individual neurons had to independently learn to associate the inputs they received from the network with the corresponding target outputs derived from the associative memory sequence. The neurons learned these input-output associations by adjusting the weights of their input connections, $J_{ij}$ (weight of connection from neuron *j* to neuron *i*). In contrast to previous studies, we accounted for the fact that learning in the brain is accompanied by several sources of errors and noise. Within the model, these sources are divided into three categories (Fig. 1*A*, orange lightning signs): (1) input spiking errors, or errors in $X^{\mu }$, (2) synaptic noise, or noise in $J_{ij}$, and (3) intrinsic noise, which combines all other sources of noise affecting the neurons’ postsynaptic potentials (PSPs). The last category includes background synaptic activity and the stochasticity of ion channels. In the model, this category is equivalent to noise in the neurons’ thresholds of firing, *h_i_* (for neuron *i*). In the following, asterisks are used to denote quantities containing errors or noise (e.g., $X^{*\mu }$), whereas symbols without asterisks represent the mean (for *h_i_* and *J_ij_*) or target (for $X^{\mu }$) values. The three types of errors and noise collectively corrupt the neurons’ outputs, $X_{i}^{*\mu +1}=\theta \left(\sum_{j=1}^{N}J_{ij}^{*}X_{j}^{*\mu }-h_{i}^{*}\right)$, making them different from the target outputs, $X_{i}^{\mu +1}$. Here, *θ* denotes the Heaviside step-function. As the probability of action potential failure in neocortical axons is small (Cox et al., 2000), we assumed that no additional errors affect the neurons’ outputs before they become inputs for the next time step.

The target neuron activities (e.g., binary scalar $X_{i}^{\mu }$) were independently drawn from neuron-dependent Bernoulli probability distributions: 0 with probability 1 – *f_i_* and 1 with probability *f_i_*. Spiking errors in neuron activity states were introduced with the Bernoulli trials by making independent and random 1–0 changes with probabilities $P\left(X_{i}^{*\mu }=0|X_{i}^{\mu }=1\right)\equiv p_{i}^{-}$ for spike failures and 0–1 changes with probabilities $P\left(X_{i}^{*\mu }=1|X_{i}^{\mu }=0\right)\equiv p_{i}^{+}$ for erroneous spikes. Without loss of generality, we assumed that these two types of spiking errors are balanced, $f_{i}p_{i}^{-}=\left(1-f_{i}\right)p_{i}^{+}$, and do not affect the neuron’s firing probability, *f_i_*. This relation allowed us to describe both types of spiking errors in terms of the neuron’s overall spiking error probability, $r_{i}=f_{i}p_{i}^{-}+\left(1-f_{i}\right)p_{i}^{+}$, i.e., $p_{i}^{+}=\frac{r_{i}}{2\left(1-f_{i}\right)}$ and $p_{i}^{-}=\frac{r_{i}}{2f_{i}}$.

To describe synaptic noise, we followed the basic model of quantal synaptic transmission (Del Castillo and Katz, 1954) and assumed that the variance of a given connection weight, $J_{ij}^{*}$, is proportional to its mean, $\mathrm{var}\left(J_{ij}^{*}\right)=\frac{h_{i}\beta_{syn,\;i}}{N}\left|J_{ij}\right|$. The dimensionless coefficient *β_syn, i_* is referred to as the synaptic noise strength of neuron *i*, and the factor of *h_i_*/*N* was introduced for convenience. We assumed that the intrinsic noise is Gaussian distributed across trials with the mean $〈h_{i}^{*}〉=h_{i}$ and variance $\mathrm{var}\left(h_{i}^{*}\right)=\frac{h_{i}^{2}\beta_{int,\;i}^{2}}{N}$. Here, $\beta_{int,\;i}$ is a dimensionless coefficient called the intrinsic noise strength of neuron *i*, and, as before, a factor of $h_{i}^{2}/N$ was introduced for convenience.

Similar to Chapeton et al. (2015), two biologically inspired constraints were imposed on the learning process. First, the *l*_1_-norm of input connection weights of each neuron was fixed during learning, $\frac{1}{N}\sum_{j=1}^{N}\left|J_{ij}\right|=w_{i}$. Here, parameter *w_i_* is referred to as the average absolute connection weight of neuron *i*. Second, the signs of output connection weights of every neuron (inhibitory or excitatory) were fixed during learning, $J_{ij}g_{j}\geq 0$. In these *N*^2^ inequalities, parameter $g_{j}=1$ if neuron *j* is excitatory and –1 if it is inhibitory. Biological motivations for these constraints were previously discussed (Chapeton et al., 2015).

Individual neurons (e.g., neuron *i*) learned independently to associate noisy inputs they received from the network, $X^{*\mu }$, with the corresponding target outputs (not corrupted by noise) derived from the associative memory sequence, $X_{i}^{\mu +1}$. Neuron *i* is said to have learned the presented set of associations successfully if, in the presence of input spiking errors, synaptic and intrinsic noise, the fractions of its erroneous and failed spikes do not exceed its assigned spiking error probabilities, $p_{i}^{+}$ and $p_{i}^{-}$ (Fig. 1*B*). The above-described model for neuron *i* can be summarized as follows:

(1) $$\begin{array}{l} P\left(\theta \left(\sum_{j=1}^{N}J_{ij}^{*}X_{j}^{*\mu }-h_{i}^{*}\right)=0|X_{i}^{\mu +1}=1\right)\leq \frac{r_{i}}{2f_{i}};\;\\ \mu =1,...,m,\;i,j=1,...,N\\ P\left(\theta \left(\sum_{j=1}^{N}J_{ij}^{*}X_{j}^{*\mu }-h_{i}^{*}\right)=1|X_{i}^{\mu +1}=0\right)\leq \frac{r_{i}}{2\left(1-f_{i}\right)}\\ P\left(X_{i}^{*\mu }=0|X_{i}^{\mu }=1\right)=\frac{r_{i}}{2f_{i}};\;P\left(X_{i}^{*\mu }=1|X_{i}^{\mu }=0\right)=\frac{r_{i}}{2\left(1-f_{i}\right)};\;P\left(X_{i}^{\mu }=1\right)=f_{i}\\ 〈J_{ij}^{*}〉=J_{ij};\;\mathrm{var}\left(J_{ij}^{*}\right)=\frac{h_{i}\beta_{syn,\;i}}{N}\left|J_{ij}\right|\\ 〈h_{i}^{*}〉=h_{i};\;\mathrm{var}\left(h_{i}^{*}\right)=\frac{h_{i}^{2}\beta_{int,\;i}^{2}}{N}\\ \frac{1}{N}\sum_{j=1}^{N}\left|J_{ij}\right|=w_{i}\\ J_{ij}g_{j}\geq 0 \end{array}$$

We note that, depending on the loaded associative memory sequence, Equation 1 may have multiple solutions if the learning problem faced by the neuron is feasible or no solution if the problem is not feasible. The neuron’s success probability in learning associative sequences of a given length is defined as the average of such binary outcomes (Fig. 1*C*). It is a decreasing function of the memory load and levels of errors and noise.

At the network level, the described associative memory storage model is governed by the network-related parameters *N* and {*g_i_*}, the memory load *m*/*N*, and the neuron-related parameters {*h_i_*}, {*w_i_*}, $\left\{f_{i}\right\}$, $\left\{r_{i}\right\}$, $\left\{\beta_{syn,\;i}\right\}$, and $\left\{\beta_{int,\;i}\right\}$. The task is to find connection weights, $\left\{J_{ij}\right\}$, that satisfy the requirements of Equation 1 for all neurons. In the following, we examine the properties of associative networks composed of inhibitory and excitatory neurons governed by identical ($h_{i}=h$, $w_{i}=w$, $f_{i}=f$, $r_{i}=r$, $\beta_{int,\;i}=\beta_{int}$, and $\beta_{syn,\;i}=\beta_{syn}$) and distributed neuron-related parameters. We refer to these networks as homogeneous and heterogeneous.

### Single-neuron model of associative memory storage in the presence of errors and noise

Each neuron in the network (e.g., neuron *i*) receives *N_inh_* inhibitory and (*N − N_inh_*) excitatory input connections (Fig. 1*A*) and independently from other neurons attempts to solve the problem outlined by Equation 1. This single-neuron learning problem was solved with the replica method in the limit of infinite network size (Edwards and Anderson, 1975; Sherrington and Kirkpatrick, 1975) and numerically with nonlinear optimization and perceptron-type learning rule for large but finite networks. In contrast to previous studies (Gardner, 1988; Gardner and Derrida, 1988; Brunel et al., 2004; Chapeton et al., 2012, 2015; Brunel, 2016; Rubin et al., 2017; Zhang et al., 2019b), the solution explicitly accounts for several distinct sources of errors and noise present during learning and incorporates two biologically inspired constraints on connectivity.

To simplify the notation in this single-neuron learning problem, in the following, we redefine the variables related to the neuron’s output, $X_{i}^{\mu +1}$ with $y^{\mu }$, $f_{i}$ with $f_{out}$, $r_{i}$ with $r_{out}$, and drop index *i*. The model is then summarized like so:

(2) $$\begin{array}{l} \theta \left(\sum_{j=1}^{N}J_{j}^{*}X_{j}^{*\mu }-h^{*}\right)=y^{*\mu };\;\mu =1,...,m,\;j=1,...,N\\ P\left(X_{j}^{*\mu }=0|X_{j}^{\mu }=1\right)=\frac{r_{j}}{2f_{j}};\;P\left(X_{j}^{*\mu }=1|X_{j}^{\mu }=0\right)=\frac{r_{j}}{2\left(1-f_{j}\right)};\;P\left(X_{j}^{\mu }=1\right)=f_{j}\\ P\left(y^{*\mu }=0|y^{\mu }=1\right)\leq \frac{r_{out}}{2f_{out}};\;P\left(y^{*\mu }=1|y^{\mu }=0\right)\leq \frac{r_{out}}{2\left(1-f_{out}\right)};\;P\left(y^{\mu }=1\right)=f_{out}\\ 〈J_{j}^{*}〉=J_{j};\;\mathrm{var}\left(J_{j}^{*}\right)=\frac{h\beta_{syn}}{N}\left|J_{j}\right|\\ 〈h^{*}〉=h;\;\mathrm{var}\left(h^{*}\right)=\frac{h^{2}\beta_{int}^{2}}{N}\\ \frac{1}{N}\sum_{j=1}^{N}\left|J_{j}\right|=w\\ J_{j}g_{j}\geq 0 \end{array}$$

Learning in the model is accompanied by four types of errors and noise. These include presynaptic and output spiking errors, or errors in $X^{\mu }$ and $y^{\mu }$, synaptic noise, or noise in *J*, and intrinsic noise, or noise in the neuron’s threshold of firing, *h*. As before, we use asterisks to denote quantities containing errors or noise (e.g., $X^{*\mu }$), whereas variables without asterisks represent the mean (for *h* and *J_j_*) or target (for $X^{\mu }$ and $y^{\mu }$) values. The neuron is faced with the task of finding connection weights, $\left\{J_{j}\right\}$, that satisfy Equation 2 for a given set of model parameters: $N,\;m/N,\;h,\;w,\;\left\{g_{j}\right\},\;\left\{f_{j}\right\},\;f_{out},\;\left\{r_{j}\right\},\;r_{out},\;\beta_{syn},\;\beta_{int}.$

### Reformulation of the model in the large *N* limit

In the limit of large *N*, the Central Limit Theorem ensures that the neuron’s PSP, $\sum_{j=1}^{N}J_{j}^{*}X_{j}^{*\mu }$, is Gaussian distributed at every time step. Therefore, the deviation of PSP from the threshold of firing, $I^{*\mu }=\sum_{j=1}^{N}J_{j}^{*}X_{j}^{*\mu }-h^{*}$, is also Gaussian distributed with the mean and SD given by the following expressions:

(3) $$\begin{array}{l} I^{\mu }=\sum_{j=1}^{N}J_{j}\left[\left(1-\frac{r_{j}}{2f_{j}}\right)X_{j}^{\mu }+\frac{r_{j}\left(1-X_{j}^{\mu }\right)}{2\left(1-f_{j}\right)}\right]-h\\ {\left(\sigma^{\mu }\right)}^{2}=\sum_{j=1}^{N}J_{j}^{2}\left[\left(1-\frac{r_{j}}{2f_{j}}\right)\frac{r_{j}X_{j}^{\mu }}{2f_{j}}+\left(1-\frac{r_{j}}{2\left(1-f_{j}\right)}\right)\frac{r_{j}\left(1-X_{j}^{\mu }\right)}{2\left(1-f_{j}\right)}\right]\\ +\frac{h\beta_{syn}}{N}\sum_{j=1}^{N}J_{j}g_{j}\left[\left(1-\frac{r_{j}}{2f_{j}}\right)X_{j}^{\mu }+\frac{r_{j}\left(1-X_{j}^{\mu }\right)}{2\left(1-f_{j}\right)}\right]+\frac{h^{2}\beta_{int}^{2}}{N}. \end{array}$$

As a result, the inequality constraints on the probabilities of output spiking errors (Eq. 2, line three) can be expressed in terms of $I^{\mu }$ and $\sigma^{\mu }$:

(4) $$\begin{array}{l} I^{\mu }\geq \sqrt{2}{\text{erf}}^{-1}\left(1-\frac{r_{out}}{f_{out}}\right)\sigma^{\mu },\;y^{\mu }=1\\ I^{\mu }\leq -\sqrt{2}{\text{erf}}^{-1}\left(1-\frac{r_{out}}{1-f_{out}}\right)\sigma^{\mu },\;y^{\mu }=0. \end{array}$$

The above two inequalities can be combined into a single expression that must hold for a successfully learned association *μ*:

(5) $$\left(2y^{\mu }-1\right)I^{\mu }\geq \sqrt{2}\left({\text{erf}}^{-1}\left(1-\frac{r_{out}}{f_{out}}\right)y^{\mu }+{\text{erf}}^{-1}\left(1-\frac{r_{out}}{1-f_{out}}\right)\left(1-y^{\mu }\right)\right)\sigma^{\mu }$$

### Additional assumptions required for the replica calculation

Following the procedure outlined in Zhang et al. (2019b), we assumed that the model parameters $m/N,\;\left\{f_{j}\right\},\;f_{out},\;\left\{r_{j}\right\},\;r_{out},\;\beta_{syn},\;\beta_{int}$ are intensive, or of order 1 in *N*. Also, we assumed that the connection weights are inversely proportional to the system size, $\left\{J_{j}=\frac{h}{N}{\tilde{J}}_{j}\right\}$, and refer to $\left\{{\tilde{J}}_{j}\right\}$ as scaled connection weights. This particular scaling is traditionally used in associative memory models (Brunel et al., 2004), and it has been shown that in the biologically plausible high-weight regime, Nwf >> h, many model results become independent of this assumption (Zhang et al., 2019b). It follows from the sixth line of Equation 2 that $w=\frac{h}{N}\tilde{w}$, and we refer to $\tilde{w}$ as scaled average absolute connection weight.

The model, rewritten in terms of the scaled variables, contains one equality and *m *+* N* inequality constraints:

(6) $$\begin{array}{l} \left(2y^{\mu }-1\right)\left(\frac{1}{N}\sum_{j=1}^{N}{\tilde{J}}_{j}\left[\left(1-\frac{r_{j}}{2f_{j}}\right)X_{j}^{\mu }+\frac{r_{j}\left(1-X_{j}^{\mu }\right)}{2\left(1-f_{j}\right)}\right]-1\right)\\ \geq \sqrt{\frac{2}{N}}\left({\text{erf}}^{-1}\left(1-\frac{r_{out}}{f_{out}}\right)y^{\mu }+{\text{erf}}^{-1}\left(1-\frac{r_{out}}{1-f_{out}}\right)\left(1-y^{\mu }\right)\right)\\ \times {\left(\frac{1}{N}\sum_{j=1}^{N}\left[{\tilde{J}}_{j}^{2}\left(\left(1-\frac{r_{j}}{2f_{j}}\right)\frac{r_{j}X_{j}^{\mu }}{2f_{j}}+\left(1-\frac{r_{j}}{2\left(1-f_{j}\right)}\right)\frac{r_{j}\left(1-X_{j}^{\mu }\right)}{2\left(1-f_{j}\right)}\right)+\beta_{syn}{\tilde{J}}_{j}g_{j}\left(\left(1-\frac{r_{j}}{2f_{j}}\right)X_{j}^{\mu }+\frac{r_{j}\left(1-X_{j}^{\mu }\right)}{2\left(1-f_{j}\right)}\right)+\beta_{int}^{2}\right]\right)}^{\frac{1}{2}};\;\mu =1,...,m\\ \frac{1}{N}\sum_{j=1}^{N}{\tilde{J}}_{j}g_{j}=\tilde{w}\\ {\tilde{J}}_{j}g_{j}\geq 0;\;j=1,...,N\\ P\left(X_{j}^{\mu }=1\right)=f_{j};\;P\left(y^{\mu }=1\right)=f_{out}. \end{array}$$

In the following, we only consider the output spiking error probabilities in the ranges $p_{out}^{+}<f_{out}$ and $p_{out}^{-}<1-f_{out}$, which is equivalent to $r_{out}<2f_{out}\left(1-f_{out}\right)$. This is required for the stability of the replica solution.

### Replica theory solution of the model

We begin by calculating the volume of the connection weight space, $\Omega \left(\left\{X^{\mu },y^{\mu }\right\}\right)$, in which Equation 6 holds for a given set of associations, $\left\{X^{\mu },y^{\mu }\right\}$:

(7) $$\begin{array}{l} \Omega \left(\left\{X^{\mu },y^{\mu }\right\}\right)=\int \prod_{j=1}^{N}d{\tilde{J}}_{j}\prod_{j=1}^{N}\theta \left({\tilde{J}}_{j}g_{j}\right)\delta \left(\frac{1}{N}\sum_{j=1}^{N}{\tilde{J}}_{j}g_{j}-\tilde{w}\right)\prod_{\mu =1}^{m}\theta (\begin{array}{l} \left(2y^{\mu }-1\right)\times \end{array}\\ \left(\frac{1}{N}\sum_{j=1}^{N}{\tilde{J}}_{j}\left[\left(1-\frac{r_{j}}{2f_{j}}\right)X_{j}^{\mu }+\frac{r_{j}\left(1-X_{j}^{\mu }\right)}{2\left(1-f_{j}\right)}\right]-1\right)-\sqrt{\frac{2}{N}}\left({\text{erf}}^{-1}\left(1-\frac{r_{out}}{f_{out}}\right)y^{\mu }+{\text{erf}}^{-1}\left(1-\frac{r_{out}}{1-f_{out}}\right)\left(1-y^{\mu }\right)\right)\times {\left(\frac{1}{N}\sum_{j=1}^{N}\left[{\tilde{J}}_{j}^{2}\left(\left(1-\frac{r_{j}}{2f_{j}}\right)\frac{r_{j}X_{j}^{\mu }}{2f_{j}}+\left(1-\frac{r_{j}}{2\left(1-f_{j}\right)}\right)\frac{r_{j}\left(1-X_{j}^{\mu }\right)}{2\left(1-f_{j}\right)}\right)+\beta_{syn}{\tilde{J}}_{j}g_{j}\left(\left(1-\frac{r_{j}}{2f_{j}}\right)X_{j}^{\mu }+\frac{r_{j}\left(1-X_{j}^{\mu }\right)}{2\left(1-f_{j}\right)}\right)+\beta_{int}^{2}\right]\right)}^{\frac{1}{2}}) \end{array}$$

The typical volume of this solution space, $\Omega_{typical}$, is defined through the averaging of $\ln\left(\Omega \left(\left\{X^{\mu },y^{\mu }\right\}\right)\right)$ over the set of associations $\left\{X^{\mu },y^{\mu }\right\}$, and is calculated by introducing *n* replica systems:

(8) $$\ln\left(\Omega_{typical}\right)={〈\ln\left(\Omega \left(\left\{X^{\mu },y^{\mu }\right\}\right)\right)〉}_{\left\{X^{\mu },y^{\mu }\right\}}=\underset{n\to 0}{\lim}\frac{{〈\Omega {\left(\left\{X^{\mu },y^{\mu }\right\}\right)}^{n}〉}_{\left\{X^{\mu },y^{\mu }\right\}}-1}{n}.$$

The quantity ${〈\Omega {\left(\left\{X^{\mu },y^{\mu }\right\}\right)}^{n}〉}_{\left\{X^{\mu },y^{\mu }\right\}}$ can be rewritten as a single multidimensional integral and calculated by following a previously established procedure (Zhang et al., 2019b). Below, we only provide the main steps of this calculation, additional details can be found in Zhang et al. (2020):

(9) $$\begin{array}{l} \ln\left(\Omega_{typical}\right)=N\left(\left(2z+\eta \tilde{w}\right)\sqrt{t}+\tau \kappa t-\frac{{\left(D_{out}^{+}+D_{out}^{-}\right)}^{2}}{2{\left(u_{+}+u_{-}\right)}^{2}}\left(\varepsilon -\delta \right)\kappa t+\alpha G_{E}\left(u_{+},u_{-},\varepsilon \right)+G_{S}\left(\eta ,t,\tau ,z,\delta \right)\right)\\ G_{E}\left(u_{+},u_{-},\varepsilon \right)=\int_{-\infty }^{\infty }\frac{e^{-x^{2}}}{\sqrt{\pi }}dx\left(f_{out}\ln\left(\text{erfc}\left(\frac{u_{-}-x}{\sqrt{\varepsilon }}\right)\right)+(1-f_{out})\ln\left(\text{erfc}\left(\frac{u_{+}-x}{\sqrt{\varepsilon }}\right)\right)\right)-\ln2\\ G_{S}\left(\eta ,t,\tau ,z,\delta \right)=\frac{1}{N}\sum_{j=1}^{N}\int_{-\infty }^{\infty }\frac{e^{-x^{2}}}{\sqrt{\pi }}dx\ln\left(e^{-\tau t\beta_{int}^{2}}\frac{\sqrt{\pi }e^{{\left(\frac{\eta +2zg_{j}f_{j}+\tau \sqrt{t}\beta_{syn}f_{j}-2x\sqrt{C_{j}}}{2\sqrt{C_{j}\delta +B_{j}\tau }}\right)}^{2}}}{2\sqrt{C_{j}\delta t+B_{j}\tau t}}\text{erfc}\left(\frac{\eta +2zg_{j}f_{j}+\tau \sqrt{t}\beta_{syn}f_{j}-2x\sqrt{C_{j}}}{2\sqrt{C_{j}\delta +B_{j}\tau }}\right)\right)\\ B_{j}=r_{j}\left(1-\frac{r_{j}}{4f_{j}\left(1-f_{j}\right)}\right);\;\;C_{j}=f_{j}\left(1-f_{j}\right){\left(1-\frac{r_{j}}{2f_{j}\left(1-f_{j}\right)}\right)}^{2};\;\;D_{out}^{+}=\sqrt{2}{\text{erf}}^{-1}\left(1-\frac{r_{out}}{1-f_{out}}\right);\;\;D_{out}^{-}=\sqrt{2}{\text{erf}}^{-1}\left(1-\frac{r_{out}}{f_{out}}\right). \end{array}$$

The nine latent variables, *u_+_*, *u_-_*, *κ*, *ε*, *η*, *t*, *τ*, *z*, and *δ* are defined by the position of the maximum of $\ln\left(\Omega_{typical}\right)$. They can be obtained by solving the following system of nine equations:

(10) $$\begin{array}{l} \alpha \frac{\partial G_{E}\left(u_{+},u_{-},\varepsilon \right)}{\partial u_{+}}+\frac{{\left(D_{out}^{+}+D_{out}^{-}\right)}^{2}}{{\left(u_{+}+u_{-}\right)}^{3}}\left(\varepsilon -\delta \right)\kappa t=0;\;\alpha \frac{\partial G_{E}\left(u_{+},u_{-},\varepsilon \right)}{\partial u_{-}}+\frac{{\left(D_{out}^{+}+D_{out}^{-}\right)}^{2}}{{\left(u_{+}+u_{-}\right)}^{3}}\left(\varepsilon -\delta \right)\kappa t=0\\ \tau t-\frac{{\left(D_{out}^{+}+D_{out}^{-}\right)}^{2}}{2{\left(u_{+}+u_{-}\right)}^{2}}\left(\varepsilon -\delta \right)t=0;\;\alpha \frac{\partial G_{E}\left(u_{+},u_{-},\varepsilon \right)}{\partial \varepsilon }-\frac{{\left(D_{out}^{+}+D_{out}^{-}\right)}^{2}}{2{\left(u_{+}+u_{-}\right)}^{2}}\kappa t=0\\ \frac{\partial G_{S}\left(\eta ,t,\tau ,z,\delta \right)}{\partial \eta }+\tilde{w}\sqrt{t}=0;\;\frac{\partial G_{S}\left(\eta ,t,\tau ,z,\delta \right)}{\partial t}+\frac{\left(2z+\eta \tilde{w}\right)}{2\sqrt{t}}+\tau \kappa -\frac{{\left(D_{out}^{+}+D_{out}^{-}\right)}^{2}}{2{\left(u_{+}+u_{-}\right)}^{2}}\left(\varepsilon -\delta \right)\kappa =0\\ \frac{\partial G_{S}\left(\eta ,t,\tau ,z,\delta \right)}{\partial \tau }+\kappa t=0;\;\frac{\partial G_{S}\left(\eta ,t,\tau ,z,\delta \right)}{\partial z}+2\sqrt{t}=0;\;\frac{\partial G_{S}\left(\eta ,t,\tau ,z,\delta \right)}{\partial \delta }+\frac{{\left(D_{out}^{+}+D_{out}^{-}\right)}^{2}}{2{\left(u_{-}+u_{+}\right)}^{2}}\kappa t=0\\ \kappa \geq 0;\;\varepsilon \geq 0;\;u_{+}+u_{-}\geq 0. \end{array}$$

The three inequality constraints in the last line of Equation 10 ensure that the solution is physical.

### Replica theory solution at critical capacity

With an increasing number of associations *m*, $\Omega_{typical}$ shrinks and approaches zero at the maximum (critical) capacity of the neuron, $\alpha_{c}=\frac{m_{c}}{N}$. In this limit, $\left(q_{0}-q\right)$ goes to zero and Equation 10 can be expanded asymptotically in terms of 1/ε and 1/δ. After replacing $\tau \sqrt{t}$ with y, δ/τ with *x*, and eliminating variables, ε, t, κ, τ, and δ, we arrived at the final system of six equations and one inequality. This system contains six latent variables $u_{\pm }$, x, η, y, and z which determine the critical capacity of the neuron, $\alpha_{c}$:

(11) $$\begin{array}{l} \left\{ \begin{array}{l} (1-f_{out})F\left(u_{+}\right)-f_{out}F\left(u_{-}\right)=0\\ x=\frac{4\left(u_{-}+u_{+}\right)}{{\left(D_{out}^{+}+D_{out}^{-}\right)}^{2}}\frac{f_{out}E\left(u_{-}\right)+(1-f_{out})E\left(u_{+}\right)}{f_{out}F\left(u_{-}\right)+(1-f_{out})F\left(u_{+}\right)}\\ \frac{1}{N}\sum_{j=1}^{N}\frac{\sqrt{C_{j}}}{C_{j}x+B_{j}}F\left(-\frac{\eta +2zf_{j}g_{j}+y\beta_{syn}f_{j}}{2\sqrt{C_{j}}}\right)=2\tilde{w}y\\ \frac{1}{N}\sum_{j=1}^{N}\frac{f_{j}g_{j}\sqrt{C_{j}}}{C_{j}x+B_{j}}F\left(-\frac{\eta +2zf_{j}g_{j}+y\beta_{syn}f_{j}}{2\sqrt{C_{j}}}\right)=2y\\ \frac{1}{N}\sum_{j=1}^{N}\frac{C_{j}}{{\left(C_{j}x+B_{j}\right)}^{2}}\left(\frac{B_{j}}{2}-\frac{{\left(u_{-}+u_{+}\right)}^{2}}{{\left(D_{out}^{+}+D_{out}^{-}\right)}^{2}}C_{j}\right)D\left(-\frac{\eta +2zf_{j}g_{j}+y\beta_{syn}f_{j}}{2\sqrt{C_{j}}}\right)=\beta_{int}^{2}y^{2}-y\tilde{w}\eta -2yz\\ \frac{1}{N}\sum_{j=1}^{N}\frac{\beta_{syn}f_{j}\sqrt{C_{j}}}{C_{j}x+B_{j}}F\left(-\frac{\eta +2zf_{j}g_{j}+y\beta_{syn}f_{j}}{2\sqrt{C_{j}}}\right)=2\tilde{w}\eta +4z-4\beta_{int}^{2}y\\ u_{+}+u_{-}\geq 0 \end{array}\right.\\ \alpha_{c}=x^{2}\frac{f_{out}D\left(u_{-}\right)+(1-f_{out})D\left(u_{+}\right)}{{\left(f_{out}E\left(u_{-}\right)+(1-f_{out})E\left(u_{+}\right)\right)}^{2}}\frac{1}{N}\sum_{j=1}^{N}\frac{C_{j}^{2}}{{\left(C_{j}x+B_{j}\right)}^{2}}D\left(-\frac{\eta +2zf_{j}g_{j}+y\beta_{syn}f_{j}}{2\sqrt{C_{j}}}\right). \end{array}$$

Functions *E*, *F*, and *D* in Equation 11 are defined as follows:

(12) $$\begin{array}{l} E\left(x\right)=\frac{1}{2}\left(1+\text{erf}\left(x\right)\right)\\ F\left(x\right)=\frac{1}{\sqrt{\pi }}e^{-x^{2}}+x\left(1+\text{erf}\left(x\right)\right)\\ D\left(x\right)=xF\left(x\right)+E\left(x\right) \end{array}$$

We note that Equation 11 contains as a limiting case the solution described in Brunel et al. (2004), where a simplified version of the model presented here was solved by minimizing the probability of output spiking errors for a given intrinsic noise strength. Equation 11 expands that result to account for additional features such as the homeostatic constraint, learning by inhibitory inputs, heterogeneity of inputs, synaptic noise, input and output spiking errors.

### Distribution of input weights at critical capacity

Connection probabilities, *P^con^*, probability densities of non-zero input weights, *p^PSP^*, and average weights of these inputs, $〈\tilde{J}〉$, at critical capacity were calculated as previously described (Zhang et al., 2019b). The result depends on the latent variables of Equation 11:

(13) $$\begin{array}{l} P_{j}^{con}=E\left(-\frac{\eta +2zf_{j}g_{j}+y\beta_{syn}f_{j}}{2\sqrt{C_{j}}}\right)\\ p_{j}^{PSP}\left(\tilde{J}\right)=\frac{\theta \left(g_{j}\tilde{J}\right)}{\sqrt{2\pi }\sigma_{j}\tilde{w}E\left(-\frac{\eta +2zf_{j}g_{j}+y\beta_{syn}f_{j}}{2\sqrt{C_{j}}}\right)}e^{-{\left(\frac{\tilde{J}}{\sqrt{2}\sigma_{j}\tilde{w}}+\frac{\eta +2zf_{j}g_{j}+y\beta_{syn}f_{j}}{2\sqrt{C_{j}}}g_{j}\right)}^{2}}\\ 〈{\tilde{J}}_{j}〉=g_{j}\sigma_{j}\tilde{w}\frac{F\left(E^{-1}\left(P_{j}^{con}\right)\right)}{\sqrt{2}P_{j}^{con}}\\ \sigma_{j}=\frac{\sqrt{C_{j}}}{\sqrt{2}\tilde{w}y\left(C_{j}x+B_{j}\right)}. \end{array}$$

A given input, *j*, has a non-infinitesimal probability of having a connection weight of zero, while its probability density for non-zero connection weights is a truncated Gaussian with a mean $〈{\tilde{J}}_{j}〉$ and SD $\sigma_{j}\tilde{w}$.

Equations 11, 13 were solved in MATLAB to produce the results for heterogeneous networks consisting of inhibitory and excitatory neurons with distributed spiking error probabilities and distributed intrinsic and synaptic noise strengths. The code is available at Zhang et al. (2019a). In both cases, the remaining model parameters were the same for all input connections (e.g., $f_{i}\equiv f$). In this case, the solutions of Equations 11, 13 depend on $\beta_{int}$ and $\beta_{syn}$ only in a combination $\beta =\sqrt{\beta_{int}^{2}+\tilde{w}\beta_{syn}f}$, referred to as the postsynaptic noise strength.

### The solution in the case of two homogeneous classes of inputs

In this case, all inputs have the same firing probability, *f_in_*, and the same spiking error probability, *r_in_*. Equation 11, 13 simplify significantly after the introduction of two new variables, $v_{\pm }=\frac{-\eta \pm 2zf_{in}-y\beta_{syn}f_{in}}{2\sqrt{C_{in}}}$:

(14) $$\begin{array}{l} \left\{ \begin{array}{l} (1-f_{out})F\left(u_{+}\right)-f_{out}F\left(u_{-}\right)=0\\ \frac{N_{inh}}{N}F\left(v_{+}\right)+\frac{N_{exc}}{N}F\left(v_{-}\right)=\frac{\sqrt{2}}{\sigma }\\ -\frac{N_{inh}}{N}F\left(v_{+}\right)+\frac{N_{exc}}{N}F\left(v_{-}\right)=\frac{\sqrt{2}}{\sigma \tilde{w}f_{in}}\\ \left({\left(u_{-}+u_{+}\right)}^{2}-2\xi \right)\left(\frac{N_{inh}}{N}D\left(v_{+}\right)+\frac{N_{exc}}{N}D\left(v_{-}\right)\right)=\frac{2\beta^{2}\zeta^{2}}{\sigma^{2}}\\ \sigma =\frac{\sqrt{2}\beta^{2}\zeta^{2}}{\left(\left(u_{-}+u_{+}\right)\frac{f_{out}E\left(u_{-}\right)+(1-f_{out})E\left(u_{+}\right)}{f_{out}F\left(u_{-}\right)+(1-f_{out})F\left(u_{+}\right)}+\xi \right)(\frac{1}{\tilde{w}f_{in}}\left(v_{+}-v_{-}\right)-\left(v_{+}+v_{-}\right))}\\ u_{+}+u_{-}>0 \end{array}\right.\\ \alpha_{c}=\frac{\sigma^{2}{\left(u_{-}+u_{+}\right)}^{2}}{2\beta^{4}\zeta^{4}}\frac{f_{out}D\left(u_{-}\right)+(1-f_{out})D\left(u_{+}\right)}{{\left(f_{out}F\left(u_{-}\right)+(1-f_{out})F\left(u_{+}\right)\right)}^{2}}{\left(\frac{1}{\tilde{w}f_{in}}\left(v_{+}-v_{-}\right)-\left(v_{+}+v_{-}\right)\right)}^{2}\times (\frac{N_{inh}}{N}D\left(v_{+}\right)+\frac{N_{exc}}{N}D\left(v_{-}\right))\\ P_{inh/exc}^{con}=E\left(v_{\pm }\right)\\ p_{inh/exc}^{PSP}\left(\tilde{J}\right)=\frac{\theta \left(\mp \hat{J}\right)}{\sqrt{2\pi }\sigma \tilde{w}E\left(v_{\pm }\right)}e^{-{\left(\frac{\tilde{J}}{\sqrt{2}\sigma \tilde{w}}\pm v_{\pm }\right)}^{2}}\\ 〈\left|{\tilde{J}}_{inh/exc}\right|〉=\frac{\tilde{w}}{2P_{inh/exc}^{con}}\frac{N}{N_{inh/exc}}\left(1\mp \frac{1}{\tilde{w}f_{in}}\right) \end{array}$$

The intrinsic and synaptic noises in Equation 14 are entirely contained within the parameter β, while the spiking error probabilities *r_in_* and *r_out_* appear only in the parameters *ξ* and *ζ*:

(15) $$\begin{array}{l} \beta =\sqrt{\beta_{int}^{2}+\tilde{w}\beta_{syn}f_{in}}\\ \xi =\frac{r_{in}\left(4f_{in}\left(1-f_{in}\right)-r_{in}\right)}{2{\left(2f_{in}\left(1-f_{in}\right)-r_{in}\right)}^{2}}{\left({\text{erf}}^{-1}\left(1-\frac{r_{out}}{1-f_{out}}\right)+{\text{erf}}^{-1}\left(1-\frac{r_{out}}{f_{out}}\right)\right)}^{2}\\ \zeta =\frac{\sqrt{2f_{in}\left(1-f_{in}\right)}}{\tilde{w}\left(2f_{in}\left(1-f_{in}\right)-r_{in}\right)}\left({\text{erf}}^{-1}\left(1-\frac{r_{out}}{1-f_{out}}\right)+{\text{erf}}^{-1}\left(1-\frac{r_{out}}{f_{out}}\right)\right) \end{array}$$

We note that in the absence of spiking errors in the input (*r_in_* = 0), Equation 14 is similar in structure to the solution of a traditional model considered by Zhang et al. (2019b; Fig. 1*D*). That model did not explicitly consider different sources of errors and noise, but instead used a generic robustness parameter *κ*, or a rescaled robustness parameter $\rho =\frac{\kappa }{w\sqrt{Nf\left(1-f\right)}}$, to ensure that memories are recalled reliably in the case when only intrinsic noise is present. Solutions to both models become identical when $r_{in}=0$ and ρ=βζ. Therefore, Equation 15 explains the nature of parameters *κ* and *ρ*, relating them to the output error probability, intrinsic and synaptic noise strengths:

(16) $$\begin{array}{l} \kappa =\frac{h}{\sqrt{2N}}\sqrt{\beta_{int}^{2}+\tilde{w}\beta_{syn}f_{in}}\left({\text{erf}}^{-1}\left(1-\frac{r_{out}}{1-f_{out}}\right)+{\text{erf}}^{-1}\left(1-\frac{r_{out}}{f_{out}}\right)\right)\\ \rho =\frac{\sqrt{\beta_{int}^{2}+\tilde{w}\beta_{syn}f_{in}}}{\tilde{w}\sqrt{2f_{in}\left(1-f_{in}\right)}}\left({\text{erf}}^{-1}\left(1-\frac{r_{out}}{1-f_{out}}\right)+{\text{erf}}^{-1}\left(1-\frac{r_{out}}{f_{out}}\right)\right) \end{array}$$

**Figure 2.**
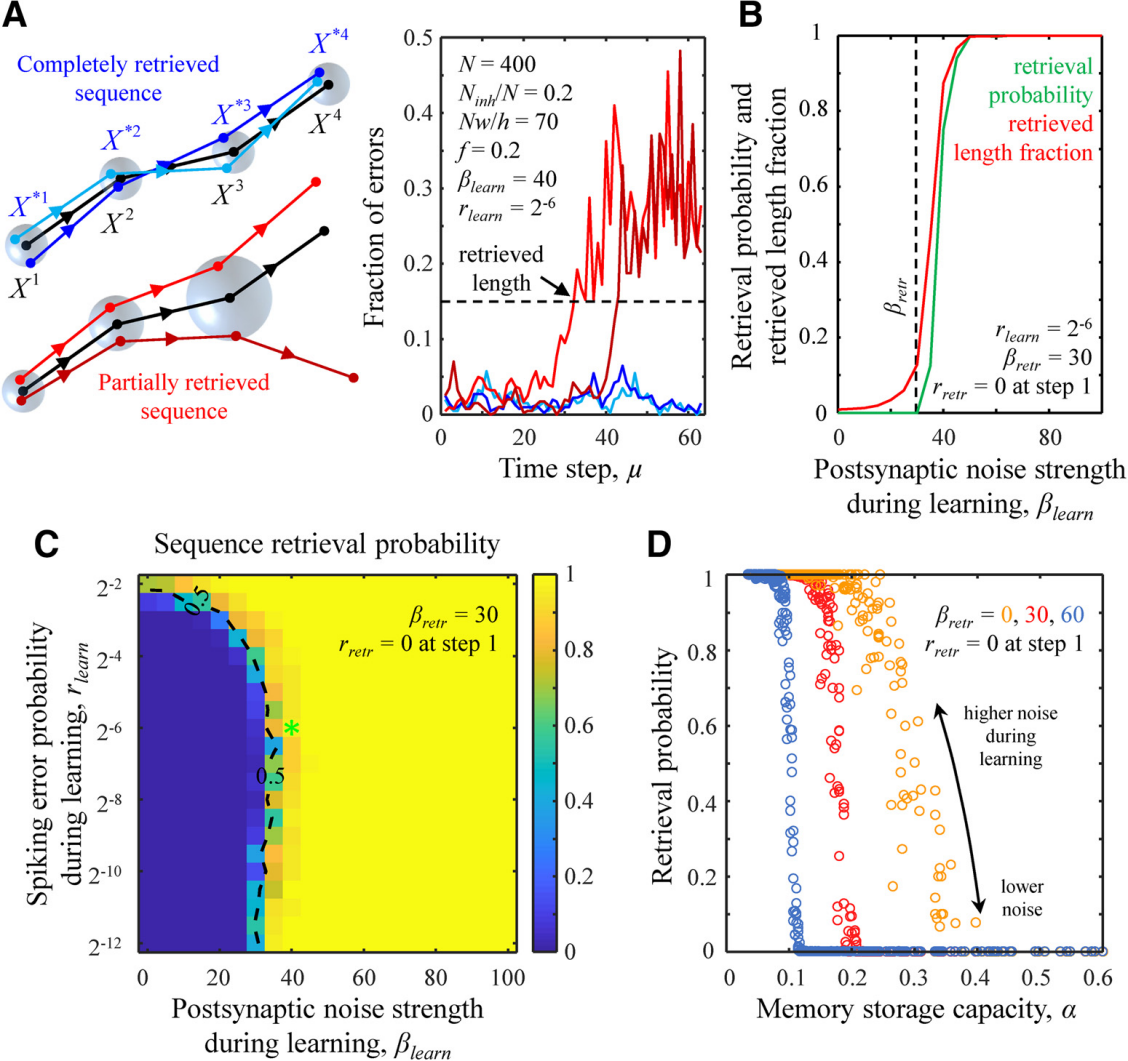
Retrieval of loaded associative memory sequences and the trade-off between capacity and reliability of loaded memories. ***A***, Illustration of memory playout during complete and partial memory retrieval (left). The target memory sequence is shown in black, while the sequences retrieved on different trials are in blue and red. Memory retrieval is incomplete when the retrieved sequence deviates significantly from the target sequence (see text for details). Radii of blue spheres illustrate the root-mean-square Euclidean distances between the retrieved and target states. The fraction of errors as a function of time step during sequence retrieval (right). Successfully retrieved sequences do not deviate from the loaded sequences by more than a threshold amount (dashed line). The parameters of the associative network are provided in the figure. The values of *β_learn_* and *r_learn_* correspond to the green asterisk from Figure 1. ***B***, The probability of successful memory retrieval (green) and the retrieved fraction of loaded sequence length (red) as a function of *β_learn_*. The postsynaptic noise strength *β_retr_* = 30 (dashed line) at every step of memory retrieval and *r_retr_* was set to 0 at the first step. ***C***, Map of retrieval probability as a function of *β_learn_* and *r_learn_*. Dashed isocontour is drawn as a guide to the eye. The location of the green asterisk is the same as in Figure 1*F*. ***D***, The trade-off between memory retrieval probability and *α*. Individual points correspond to all values of *β_learn_* and *r_learn_* considered in ***C***. Higher errors and noise during learning result in lower *α* and higher retrieval probability regardless of the noise strength during memory retrieval (different colors). The results shown in ***A–D*** were obtained with the nonlinear optimization method (see Materials and Methods). For every parameter setting, the results shown in ***B–D*** were averaged over 100 networks and 1000 retrievals of the loaded sequence in each network.

**Figure 3.**
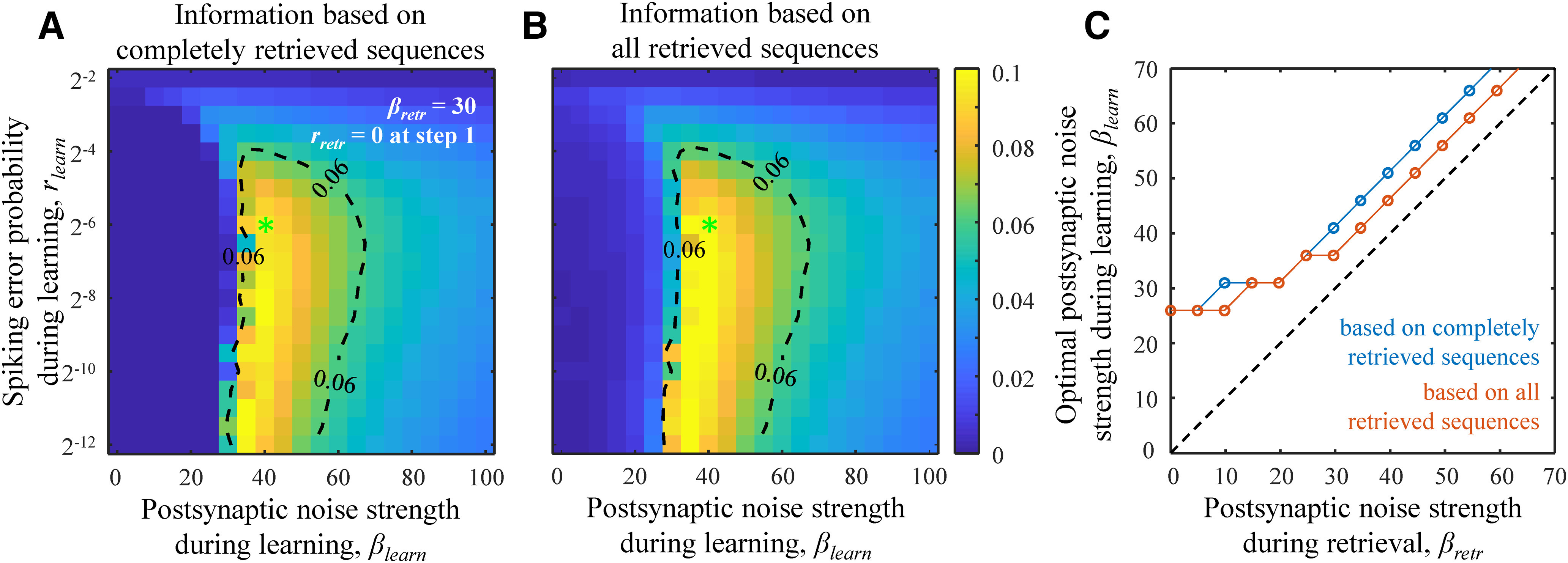
Postsynaptic noise during learning is required for optimal retrieval of stored information. ***A***, ***B***, Maps of expected retrieved information per memory playout calculated based on completely retrieved sequences (***A***) and completely and partially retrieved sequence (***B***) in bits×*N*^2^ as functions of *β_learn_* and *r_learn_*. *β_retr_* = 30 at every step of memory retrieval, and *r_retr_* was set to 0 at the first step. Dashed isocontours are drawn as guides to the eye. The locations of the green asterisks are the same as in Figure 1*F*. ***C***, The maximum of retrieved information is achieved when *β_learn_* is greater than zero regardless of the value of *β_retr_*. The optimal postsynaptic noise strengths were calculated based on the averages of the results from ***A***, blue line, and ***B***, orange line, over the range of *r_learn_* values from ***A***, ***B***. All results were obtained with the nonlinear optimization method (see Materials and Methods) and averaged over 100 networks and 1000 retrievals of the loaded sequence in each network for every parameter setting.

**Figure 4.**
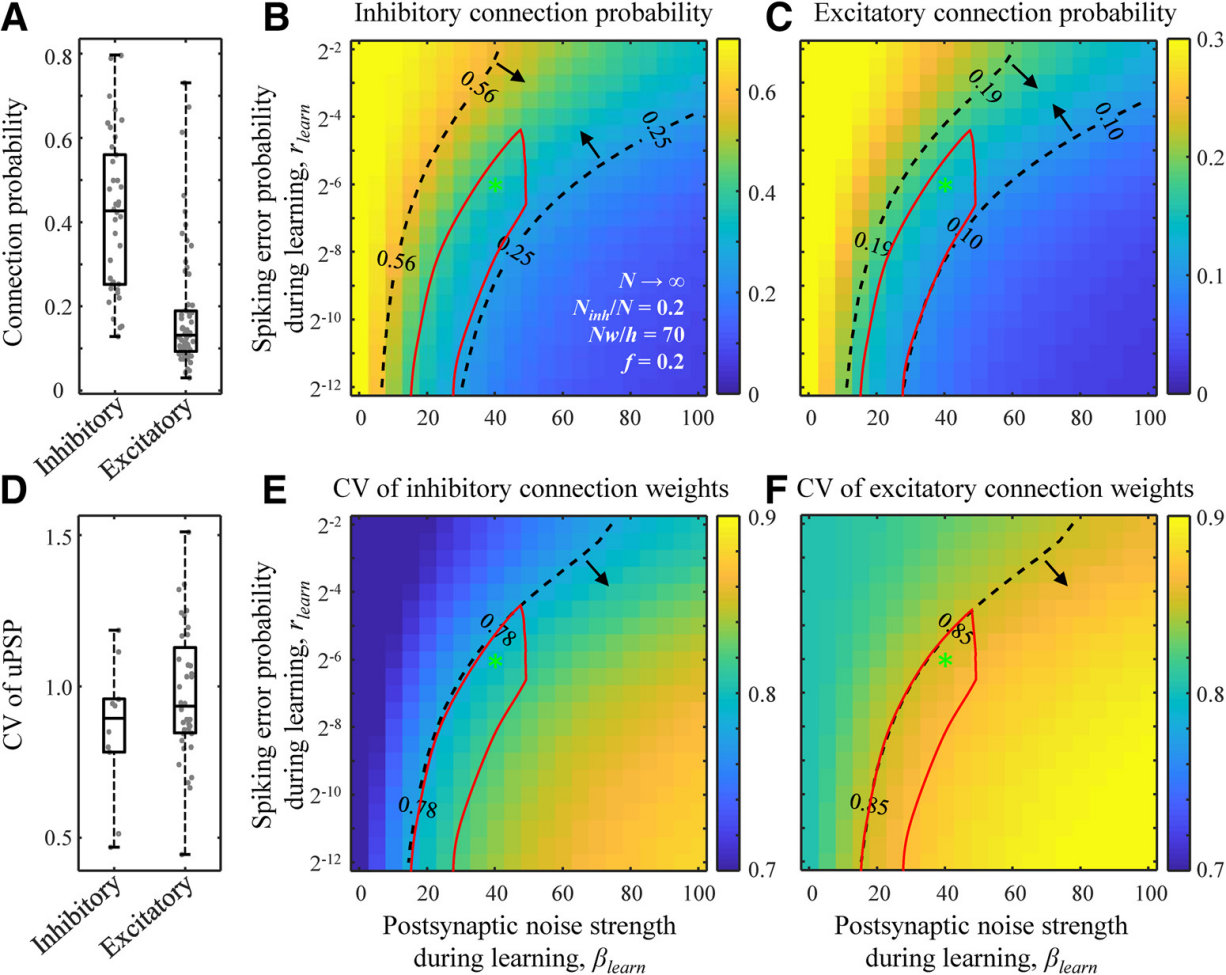
Comparison of structural properties of the model and cortical networks. ***A***, Inhibitory and excitatory connection probabilities reported in 87 studies describing 420 local cortical projections. Each dot represents the result of a single study/projection. ***B***, **C**, Maps of inhibitory and excitatory connection probabilities as functions of *β_learn_* and *r_learn_*. The results are based on the replica method (see Materials and Methods). Dashed isocontours and arrows illustrate the interquartile ranges of the experimentally observed connection probabilities from ***A***. The red contour outlines a region of parameters that is consistent with all structural and dynamical measurements in cortical networks considered in this study. The locations of the green asterisks are the same as in Figure 1*F*. ***D****–****F***, Same for the CV of non-zero inhibitory and excitatory connection weights. ***A***, ***D*** were adapted from Zhang et al. (2019b).

**Figure 5.**
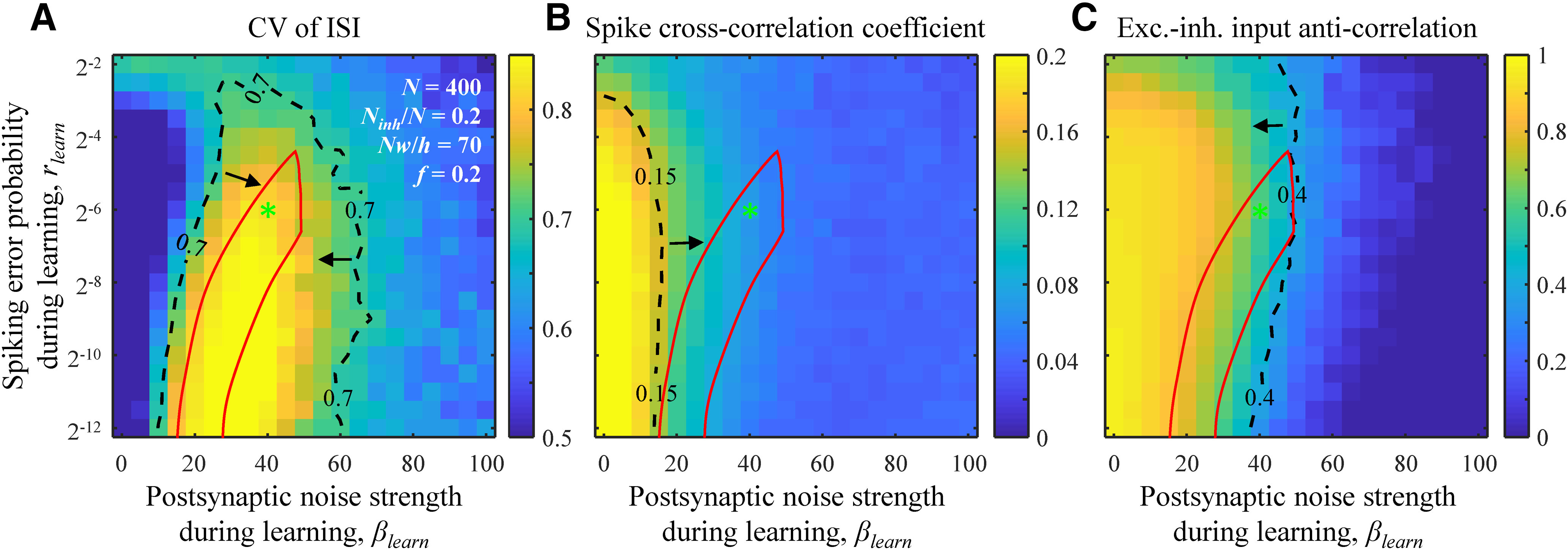
Comparison of dynamical properties of the model and cortical networks. ***A***, The CV of ISI for spontaneous (not learned) activity as a function of *β_learn_* and *r_learn_*. Dashed isocontour and arrows demarcate a region of CV values that is in general agreement with experimental measurements. ***B***, Same for the cross-correlation coefficient of neuron spike trains. ***C***, Same for the anti-correlation coefficient of inhibitory and excitatory postsynaptic inputs to a neuron. The red contour outlines a region of parameters that is consistent with the considered structural and dynamical measurements. The locations of the green asterisk are the same as in Figure 1*F*. All results were obtained with the nonlinear optimization method (see Materials and Methods) and averaged over 100 networks and 100 runs for each network for every parameter setting.

**Figure 6.**
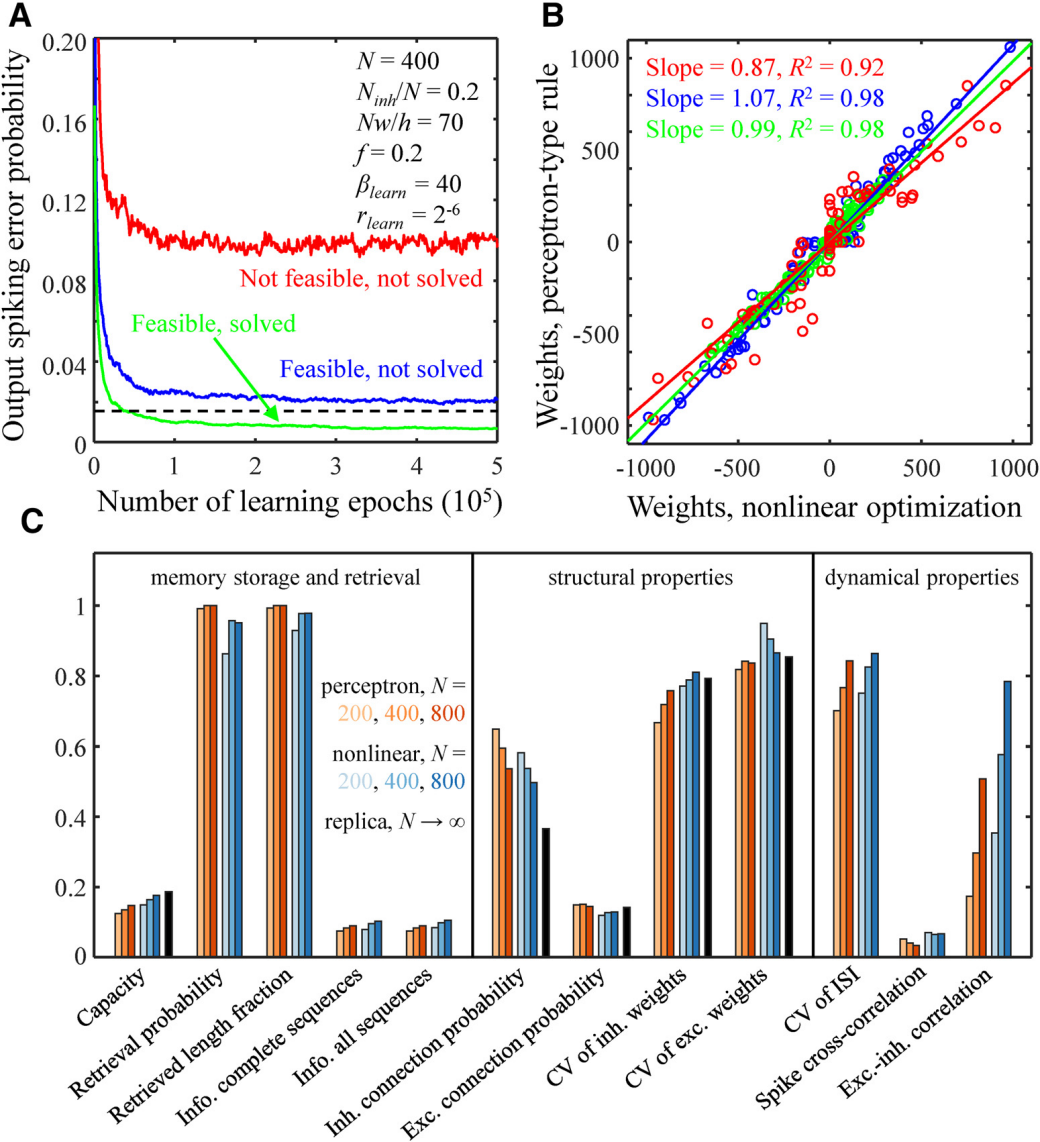
Comparison of solutions obtained with the perceptron-type learning rule, nonlinear optimization, and replica method. ***A***, Output error probability as a function of the number of learning epochs for the perceptron-type learning rule. The black dashed line indicates the target output error probability. Results for three different cases are shown: a not feasible problem (red line), a feasible problem which was not solved with the perceptron-type learning rule (blue line), and a feasible problem which was solved with the perceptron-type learning rule (green line). The parameters of the associative network are provided in the figure. The values of *β_learn_* and *r_learn_* correspond to the green asterisk from Figure 1*F*. ***B***, Comparisons of connection weights obtained with the perceptron-type learning rule and nonlinear optimization for the three cases shown in ***A***. Straight lines are the best linear fits. ***C***, Comparisons of memory storage capacity, retrieval, structural, and dynamical properties of networks of *N *=* *200, 400, and 800 neurons obtained with the perceptron-type learning rule (red colors) and nonlinear optimization (blue colors). The memory storage capacity and structural properties calculated with the replica method in the *N* → ∞ limit are shown in black.

Numerical solution of Equations 14, 15 shows that the critical capacity (Fig. 1) and probabilities of inhibitory and excitatory connections decrease with *β_int_*, *β_syn_*, and *r_in_*, and increase with *r_out_*. This is consistent with previous results (Brunel et al., 2004; Zhang et al., 2019b) showing that the critical capacity and connection probabilities are decreasing functions of *ρ*. The averages and SDs of inhibitory and excitatory connection weight magnitudes exhibit an opposite dependence on errors and noise, which is also consistent with the results of these studies. For homogeneous associative networks, we set *r_in_* = *r_out_* ≡ *r* and *f_in_* = *f_out_* ≡ *f* in Equations 14, 15, as these parameters must be the same for all neurons in the network. This does not alter the trend of the results related to *β*, but the dependence on *r* becomes more complex (Fig. 1*F*). Figures 2–6 show the results for homogeneous networks as functions of *β* and *r*.

The average weights of non-zero inhibitory and excitatory connections are uniquely determined by $\tilde{w}$, $P_{inh/exc}^{con}$, $N_{inh/exc}/N$, and *f_in_* (Eq. 14, last line). This result is obtained from the functional form of the input weight distribution, but it also follows from the fact that the input connection weights are homeostatically constrained (Eq. 6, second line) and, at critical capacity, the neuron operates in a balanced regime in which inhibitory and excitatory currents are anti-correlated and largely cancel each other out (Rubin et al., 2017). Experimentally, it has been shown that inhibitory postsynaptic currents are larger in magnitude than excitatory (Atallah and Scanziani, 2009; Salkoff et al., 2015; Feng et al., 2019). Although Equation 14 derived in the *N* → ∞ limit yield a small positive or zero average postsynaptic input (high-weight regime), associative networks of finite-size loaded with memories to capacity show a trend consistent with the experimental measurements (Zhang et al., 2019b).

### Numerical solution of the model with nonlinear optimization

For a finite number of inputs, the solution to the problem outlined in Equation 6 was obtained numerically. To that end, we made the problem feasible by introducing a slack variable $s^{\mu }\geq 0$ for every association and chose the solution that minimizes the sum of these variables:

(17) $$\begin{array}{l} \underset{\left\{{\tilde{J}}_{j}\right\}}{\arg\min}\left(\sum_{\mu =1}^{m}s^{\mu }\right)\\ \left(2y^{\mu }-1\right)\left(\frac{1}{N}\sum_{j=1}^{N}{\tilde{J}}_{j}\left[\left(1-\frac{r_{j}}{2f_{j}}\right)X_{j}^{\mu }+\frac{r_{j}\left(1-X_{j}^{\mu }\right)}{2\left(1-f_{j}\right)}\right]-1\right)\\ \geq -s^{\mu }+\frac{D_{out}^{-}y^{\mu }+D_{out}^{+}\left(1-y^{\mu }\right)}{\sqrt{N}}\times {\left(\frac{1}{N}\sum_{j=1}^{N}\left[{\tilde{J}}_{j}^{2}\left(\left(1-\frac{r_{j}}{2f_{j}}\right)\frac{r_{j}X_{j}^{\mu }}{2f_{j}}+\left(1-\frac{r_{j}}{2\left(1-f_{j}\right)}\right)\frac{r_{j}\left(1-X_{j}^{\mu }\right)}{2\left(1-f_{j}\right)}\right)+\beta_{syn}{\tilde{J}}_{j}g_{j}\left(\left(1-\frac{r_{j}}{2f_{j}}\right)X_{j}^{\mu }+\frac{r_{j}\left(1-X_{j}^{\mu }\right)}{2\left(1-f_{j}\right)}\right)+\beta_{int}^{2}\right]\right)}^{\frac{1}{2}};\;\mu =1,...,m\\ \frac{1}{N}\sum_{j=1}^{N}{\tilde{J}}_{j}g_{j}=\tilde{w}\\ {\tilde{J}}_{j}g_{j}\geq 0;\;j=1,...,N\\ s^{\mu }\geq 0;\;\mu =1,...,m. \end{array}$$

Equation 17 were solved by using the *fmincon* function of MATLAB and the results are shown in Figures 2, 3, 5, 6. The *fmincon* function utilizes the interior-point technique for finding solutions to constrained nonlinear optimization problems (Byrd et al., 1999, 2000). The code is available at Zhang et al. (2019a).

### Numerical solution of the model with a perceptron-type learning rule

In addition to the replica and nonlinear optimization solutions, a biologically more plausible online solution of Equation 17 was devised by approximately stepping in the direction of the negative gradient of the sum of the slack variables. The latter is:

(18) $$\begin{array}{l} -\frac{\partial }{\partial {\tilde{J}}_{j}}\sum_{\mu =1}^{m}s^{\mu }=\frac{1}{N}\sum_{\mu =1}^{m}\left(2y^{\mu }-1\right)\left[\left(1-\frac{r_{j}}{2f_{j}}\right)X_{j}^{\mu }+\frac{r_{j}\left(1-X_{j}^{\mu }\right)}{2\left(1-f_{j}\right)}\right]-\frac{1}{N^{3/2}}\sum_{\mu =1}^{m}\left(D_{out}^{-}y^{\mu }+D_{out}^{+}\left(1-y^{\mu }\right)\right)\\ \times \frac{{\tilde{J}}_{j}\left(\left(1-\frac{r_{j}}{2f_{j}}\right)\frac{r_{j}X_{j}^{\mu }}{2f_{j}}+\left(1-\frac{r_{j}}{2\left(1-f_{j}\right)}\right)\frac{r_{j}\left(1-X_{j}^{\mu }\right)}{2\left(1-f_{j}\right)}\right)+\frac{1}{2}\beta_{syn}g_{j}\left(\left(1-\frac{r_{j}}{2f_{j}}\right)X_{j}^{\mu }+\frac{r_{j}\left(1-X_{j}^{\mu }\right)}{2\left(1-f_{j}\right)}\right)}{{\left(\frac{1}{N}\sum_{j=1}^{N}\left[{\tilde{J}}_{j}^{2}\left(\left(1-\frac{r_{j}}{2f_{j}}\right)\frac{r_{j}X_{j}^{\mu }}{2f_{j}}+\left(1-\frac{r_{j}}{2\left(1-f_{j}\right)}\right)\frac{r_{j}\left(1-X_{j}^{\mu }\right)}{2\left(1-f_{j}\right)}\right)+\beta_{syn}{\tilde{J}}_{j}g_{j}\left(\left(1-\frac{r_{j}}{2f_{j}}\right)X_{j}^{\mu }+\frac{r_{j}\left(1-X_{j}^{\mu }\right)}{2\left(1-f_{j}\right)}\right)+\beta_{int}^{2}\right]\right)}^{\frac{1}{2}}} \end{array}$$

The first approximation to this gradient was made by omitting the second term in the right-hand side of Equation 18. This was done because this term is smaller than the first term (for large enough *N*) and because there is no clear way of calculating it in an online, biologically plausible manner. The second approximation was made by noting that $\left[\left(1-\frac{r_{j}}{2f_{j}}\right)X_{j}^{\mu }+\frac{r_{j}\left(1-X_{j}^{\mu }\right)}{2\left(1-f_{j}\right)}\right]$ in the first term in the right-hand side of Equation 18 is the average of $X_{j}^{*\mu }$ over the spiking errors, and therefore, a stochastic estimate of this gradient direction can be made in an online manner with a perceptron-type learning step $\left(2y^{\mu }-1\right)X_{j}^{*\mu }$ (Rosenblatt, 1962). These approximations lead to the learning rule of Equation 22. Related rules, in the absence of errors, noise, or *l*_1_-norm constraint, were previously described (Brunel et al., 2004; Zhang et al., 2019b).

In numerical simulations, we trained neurons on associations presented in the order of their appearance in the associative sequence, one at a time. This constitutes one learning epoch. We set the learning rate *γ* = 0.1 and ran the algorithm until a solution was found or the maximum number of 10^6^ epochs was reached. The results of this procedure are shown in Figure 6.

### Mutual information contained in retrieved associative sequences

The mutual information contained in one successfully retrieved association $\left(X^{\mu }\to X^{\mu +1}\right)$ can be calculated as a difference of marginal and conditional entropies,

(19) $$I\left(X^{\mu };X^{\mu +1}\right)=H\left(X^{\mu +1}\right)-H\left(X^{\mu +1}|X^{\mu }\right).$$

For homogeneous networks loaded with associations consisting of random and independent network states, the two entropies reduce to:

(20) $$\begin{array}{l} H\left(X^{\mu +1}\right)=-N\left[f\log_{2}f+(1-f)\log_{2}(1-f)\right]\\ H\left(X^{\mu +1}|X^{\mu }\right)=-N[f\left(\frac{r}{2f}\log_{2}\frac{r}{2f}+\left(1-\frac{r}{2f}\right)\log_{2}\left(1-\frac{r}{2f}\right)\right)\\ +\left(1-f\right)\left(\frac{r}{2\left(1-f\right)}\log_{2}\frac{r}{2\left(1-f\right)}+\left(1-\frac{r}{2\left(1-f\right)}\right)\times \log_{2}\left(1-\frac{r}{2\left(1-f\right)}\right)\right)]. \end{array}$$

As the length of a retrieved sequence may be shorter than the length of the loaded sequence, *m*, we considered two types of retrieved information. One type is defined as the expected retrieved information per memory playout in which contributions of partially retrieved sequences are set to zero. This information is based on completely retrieved sequences only and is equal to the product of the retrieval probability (Fig. 2*C*) and *mI*. The other type of retrieved information is calculated based on completely and partially retrieved sequences and is equal to the product of the average retrieved sequence length and *I*. According to these definitions, the former is always less or equal to the latter.

### Dataset of connection probabilities and strengths in local brain circuits in mammals

To compare connection probabilities and widths of non-zero connection weight distributions in associative networks with those reported experimentally, we used the dataset published in (Zhang et al., 2019b). This dataset includes measurements reported in peer-reviewed publications since 1990 in which at least 10 pairs of neurons separated laterally by <100 μm were recorded from the same layer of the mammalian neocortex in juvenile or adult animals of either sex. The dataset includes 87 publications describing 420 local projections.

## Results

### Network model of associative memory storage in the presence of errors and noise

We examined a model network consisting of *N_inh_* inhibitory and (*N* − *N_inh_*) excitatory McCulloch and Pitts neurons (McCulloch and Pitts, 1943; Fig. 1*A*) involved in associative learning. The model is described in detail in Materials and Methods, and in this subsection, we only mention its main features. The network was designed to model a local cortical circuit (∼100 μm in size) of all-to-all potentially (structurally) connected neurons (Stepanyants and Chklovskii, 2005; Stepanyants et al., 2008). The network was presented with a task of learning a sequence of consecutive network states, $X^{1}\to X^{2}\to ...X^{m+1}$, in which $X^{\mu }$ is a binary vector representing target activities of all neurons at a time step *μ*, and the ratio *m*/*N* is referred to as the memory load. Network activity in the model was accompanied by several sources of errors and noise (Fig. 1*A*, orange lightning signs), including (1) input spiking errors, or errors in $X^{\mu }$; (2) synaptic noise, or noise in connection weights, *J_ij_* (weight of connection from neuron *j* to neuron *i*); and (3) intrinsic noise, which combines all other sources of noise affecting the neurons’ PSPs. The last category includes background synaptic activity and the stochasticity of ion channels and in the model is equivalent to noise in the neurons’ firing thresholds, *h_i_*. The three types of errors and noise collectively corrupt the neurons’ outputs making them different from the target outputs. The strengths of these errors and noise in the model are governed by parameters *r_i_*, *β_syn, i_*, and *β_int, i_*, respectively.

Individual neurons in the model learned independently to associate noisy inputs they received from the network, $X^{*\mu }$, with the corresponding target outputs (not corrupted by noise) derived from the associative memory sequence, $X_{i}^{\mu +1}$. The neurons learned such input-output associations by adjusting the weights of their input connections, $J_{ij}$, in the presence of two biologically inspired constraints (Chapeton et al., 2015). First, the average absolute weight of input connections of each neuron was kept constant, *w_i_*. Second, the output connection weights of neurons (inhibitory or excitatory) did not change signs during learning.

The described associative network model is summarized by Equation 1. It is governed by the network-related parameters *N* and *N_inh_*/*N*, the memory load *m*/*N*, and the neuron-related parameters {*h_i_*}, {*w_i_*}, $\left\{f_{i}\right\}$, $\left\{r_{i}\right\}$, $\left\{\beta_{syn,\;i}\right\}$, and $\left\{\beta_{int,\;i}\right\}$. In the following, we examine the properties of associative networks with identical and distributed neuron-related parameters. These networks are referred to as homogeneous and heterogeneous.

### Solutions of the model

Equation 1 was solved with the replica method, nonlinear optimization, and a perceptron-type learning rule (see Materials and Methods). Each of these methods has its advantages and drawbacks, and, consequently, all three methods were used in this study. The replica method (Edwards and Anderson, 1975; Sherrington and Kirkpatrick, 1975) provides an analytical solution in the *N* → ∞ limit. Though neuron networks in the brain are finite, they are thought to be large enough to have many properties that are well described by this limit (Zhang et al., 2019b). More importantly, the analytical solution of the replica method reveals the dependence of the results on combinations of network parameters that can be then explored with other methods. The downside of the replica solution is that it does not provide the full connectivity matrix, *J_ij_*, but instead gives the connectivity statistics that is insufficient to calculate all relevant network properties. Nonlinear optimization can be used to solve Equation 1. This method is fast and accurate for small networks, yielding the full connectivity matrix, but is impractical for large networks (*N* ∼ 1000). As the replica and nonlinear optimization solutions cannot be readily implemented by neural networks in the brain, we also developed a biologically more plausible perceptron-type learning rule that can be used to approximate the solution of Equation 1. Because simulations based on the perceptron-type learning rule become time-consuming at or near memory storage capacity as the solution region shrinks to a point, results for varying levels of errors and noise were obtained with the replica and nonlinear optimization methods, while the perceptron-type learning rule was used only for a biologically plausible set of parameters to confirm that all three methods lead to similar results.

In the *N* → ∞ limit, the associative memory storage problem for a neuron loaded to capacity was solved with the replica method. This solution for a neuron in a homogeneous network depends on the following combination of the intrinsic and synaptic noise strengths (see Materials and Methods):

(21) $$\beta =\sqrt{\beta_{int}^{2}+\beta_{syn}fN\frac{w}{h}}$$

This quantity is referred to as the postsynaptic noise strength. In the following, we assume that the postsynaptic noise strength, *β*, and the spiking error probability, *r*, can differ between the times of learning and memory retrieval and add subscripts “*learn*” and “*retr*” to these parameters to distinguish among the two phases.

Figure 1*C* shows that when the memory load is relatively low, the probability of successful learning by a neuron is close to 1. With increasing load, the learning problem becomes more difficult, and the success probability undergoes a smooth transition from 1 to 0. Memory load corresponding to the success probability of 0.5 is referred to as the neuron’s associative memory storage capacity, *α*. With increasing network size, *N*, the transition from successful learning to inability to accurately learn the complete memory sequence becomes sharper, and the neuron’s capacity monotonically approaches its *N* → ∞ limit, which is referred to as the critical capacity, *α_c_*. The critical capacity depends on the levels of errors and noise accompanying learning and other parameters of the model. Figure 1*D–F* illustrates the dependence of *α_c_* on the input and output spiking error probabilities and postsynaptic noise strength. As expected, because input spiking errors, intrinsic, and synaptic noise, make the learning problem more challenging, *α_c_* is a decreasing function of *r_in_* (Fig. 1*D*,*E*) and *β_learn_* (Fig. 1*D*,*F*). On the other hand, the learning problem becomes simpler with increasing *r_out_* as more output errors are tolerated, and *α_c_* is an increasing function of *r_out_* (Fig. 1*E*). For a neuron in a recurrent homogeneous network, the dependence of *α_c_* on spiking errors is more complex as *r_in_* = *r_out_* ≡ *r_learn_*, and both the input and output spiking errors of the neuron are controlled by the same parameter (Fig. 1*F*).

### The trade-off between capacity and reliability of loaded memories

Can memories, loaded into individual neurons, be successfully recalled at the network level? To answer this question, we loaded neurons in the network to capacity with associations derived from a single associative sequence by solving Equation 1. The postsynaptic noise and spiking errors during learning were set at the levels *β_learn_* and *r_learn_* (Fig. 1*F*, green asterisk). During memory retrieval, the network was initialized at the beginning of the loaded sequence, $X^{1}$, and no additional spiking errors, beyond those produced by the network at subsequent steps, were added as the memory played out. At each step of memory playout, synaptic and intrinsic noise were added independently to every connection and every neuron in the network at strengths governed by *β_retr_*.

The sequence is said to be retrieved completely if the network states during the retrieval do not deviate substantially from the target states. Otherwise, the sequence is said to be retrieved partially, and the retrieved sequence length is defined by the number of steps taken to the point where the network states begin to deviate substantially from the target states (Fig. 2*A*). In practice, there is no need to precisely define the threshold amount of deviation. This is because for large networks the fraction of errors in a retrieved network state either fluctuates around $r_{learn}\pm \sqrt{r_{learn}\left(1-r_{learn}\right)/N}$ (mean ± SD) or diverges to $2f\left(1-f\right)\pm \sqrt{2f\left(1-f\right)\left(1-2f\left(1-f\right)\right)/N}$ (expected fraction of differences between two random network states of firing probability *f*), which is significantly greater for the chosen values of parameters *r_learn_* and *f*. Figure 2*B* shows the probability of retrieving a complete loaded sequence and the fraction of retrieved sequence length for different values of *β_learn_*. It illustrates that memory sequences can be reliably retrieved if they were loaded with the postsynaptic noise strength that is slightly higher than that present during memory retrieval. Likewise, the averaged retrieved sequence length fraction increases with *β_learn_* and approaches one as *β_learn_* exceeds the noise strength present during retrieval. A similar conclusion can be drawn from Figure 2*C*, which shows the map of the retrieval probability as a function of *β_learn_* and *r_learn_*. Errors and noise during learning make memory retrieval more reliable. However, the reliability of loaded memories comes at the expense of the memory storage capacity, *α*. Figure 2*D* shows the trade-off between the retrieval probability and capacity of loaded associative memories in which higher levels of errors and noise during learning enable reliable memory retrieval but reduce *α*.

### Noise during learning is required for optimal retrieval of stored information

Figure 3*A*,*B* shows the maps of expected retrieved information per sequence playout calculated in two different ways. In the first calculation, the contribution of partially retrieved sequences to the expected retrieved information was set to zero, while in the second, partially retrieved sequences contributed in the proportion of the retrieved sequence length (see Materials and Methods). Both maps illustrate that optimal retrieval of stored information is achieved when memories are stored in the presence of noise, *β_learn_* > 0. This conclusion is independent of the postsynaptic noise strength during memory retrieval, which was set to *β_retr_* = 30 in Figure 3*A*,*B*. To illustrate this finding, we averaged the maps over the *r_learn_* dimension and determined *β_learn_* that correspond to the maxima of the retrieved information. Figure 3*C* illustrates the results of this procedure for different values of *β_retr_*, showing that the optimal *β_learn_* is greater than zero even when there is no noise during memory retrieval. The optimal *β_learn_* increases with *β_retr_*, and the two noise strengths become approximately equal in the high noise limit.

### Neuron-to-neuron connectivity in associative networks of homogeneous inhibitory and excitatory neurons

One of the most salient features of sign-constrained associative learning models, such as the one described in this study, is that finite fractions of inhibitory and excitatory connections assume zero weights at capacity (Kohler and Widmaier, 1991), mirroring the trend observed in many local cortical networks. We compared the connection probabilities (*P_con_*) and the coefficients of variation (CVs) of non-zero connection weights in associative networks at capacity to the connection probabilities and CVs of unitary PSPs (uPSPs) obtained experimentally. To that end, we used the dataset compiled in (Zhang et al., 2019b) based on 87 electrophysiological studies describing neuron-to-neuron connectivity for 420 local cortical projections (lateral distance between neurons < 100 μm). Figure 4*A* shows that the average inhibitory *P_con_* (38 studies, 9522 connections tested) is significantly larger (*p* < 10^−10^, two-sample *t* test) than the average excitatory *P_con_* (67 studies, 63,020 connections tested). Associative networks exhibit a similar trend in the entire region of considered *β_learn_* and *r_learn_* values (Fig. 4*B*,*C*). What is more, in the (*β_learn_*, *r_learn_*) parameter region demarcated with the dashed isocontours and arrows in Figure 4*B*,*C*, the model results are consistent with the middle 50% of the experimentally measured *P_con_* values for inhibitory and excitatory connections.

Figure 4*D* shows that the average CV of inhibitory uPSP (10 studies, 503 connections recorded) is slightly lower than that for excitatory (36 studies, 3956 connections recorded), and this trend is also reproduced by the associative networks in the entire region of considered *β_learn_* and *r_learn_* values (Fig. 4*E*,*F*). As before, there are (*β_learn_*, *r_learn_*) parameter regions in these maps in which the results of the model are consistent with the middle 50% of the CV of uPSP measurements for inhibitory and excitatory connections.

### Spontaneous dynamics in associative networks of homogeneous inhibitory and excitatory neurons

The model associative networks can exhibit irregular and asynchronous spiking activity like that observed in cortical networks. To analyze such spontaneous (not learned) network dynamics, we used associative networks loaded to capacity, initialized them at random states of firing probability *f *=* *0.2, and followed their activity for 1000 time steps. Because the number of available network states, which is exponential in *N*, is much larger than the number of loaded states, *αN*, the spontaneous network activity in the numerical simulations never passed through any of the loaded states.

To quantify the degree of similarity in the dynamics of the model and brain networks we compared the CV of interspike-intervals (ISIs) and the cross-correlation coefficient of spiking neuron activity in the model to those measurements obtained experimentally. Figure 5*A*, dashed isocontour, outlines (*β_learn_*, *r_learn_*) parameter region in which the model CV of ISI is consistent with the 0.7–1.1 range measured in different cortical systems (Softky and Koch, 1993; Holt et al., 1996; Buracas et al., 1998; Shadlen and Newsome, 1998; Stevens and Zador, 1998). Similarly, Figure 5*B* shows that there is a (*β_learn_*, *r_learn_*) parameter region in which the calculated spike cross-correlation coefficients are in agreement with the interquartile range of the corresponding cortical measurements, 0.04–0.15 (Cohen and Kohn, 2011). The degree of asynchrony in spontaneous spiking activity in associative networks increases with the postsynaptic noise strength, which can be explained by the decrease in connection probability (Fig. 4*B*,*C*) and, consequently, a reduction in the amount of common input to the neurons.

It was shown that irregular and asynchronous activity can result from the balance of inhibitory and excitatory postsynaptic inputs to individual cells (van Vreeswijk and Sompolinsky, 1996, 1998). In a balanced state, the magnitudes of these inputs are much greater than the threshold of firing, but, because of a high degree of anti-correlation, these inputs largely cancel, and firing is driven by fluctuations. Figure 5*C* shows a region of parameters in which neurons in the associative model function in a balanced regime. Because it is difficult to simultaneously measure inhibitory and excitatory postsynaptic inputs to a neuron, the anti-correlation of inhibitory and excitatory inputs has only been measured in nearby cells, averaging to ∼0.4 (Okun and Lampl, 2008; Graupner and Reyes, 2013). As within-cell anti-correlations are expected to be stronger than between-cell anti-correlations, 0.4 was used as a lower bound for the former (Fig. 5*C*, dashed isocontour and arrow).

The seven error-noise regions obtained based on the properties of neuron-to-neuron connectivity (Fig. 4) and network dynamics (Fig. 5) have a non-empty intersection (Figs. 4, 5, red contour). In this biologically plausible region of parameters, the considered properties of the associative networks are consistent with the corresponding experimental measurements. This observation suggests that *β_learn_* must lie in the 20–50 range and *r_learn_* must be <0.06. While we are not aware of direct experimental measurements of these parameters, the low value of *r_learn_* is in qualitative agreement with the reliability of firing patterns evoked by time-varying stimuli *in vivo* (Buracas et al., 1998) and *in vitro* (Mainen and Sejnowski, 1995).

### Solution of the model with a perceptron-type learning rule

As the replica and nonlinear optimization solutions of Equation 1 cannot be easily implemented by neural networks in the brain, we set out to develop a biologically more plausible online solution to the associative learning problem. The following perceptron-type learning rule was devised to approximate the solution of Equation 1 (see Materials and Methods). At each learning step, e.g., *μ*, a neuron receives an input containing spiking errors, $X^{*\mu }$, combines it with synaptic and intrinsic noise, and produces an output corrupted by noise, $y^{*\mu }=\theta \left(\sum_{j=1}^{N}J_{j}^{*}X_{j}^{*\mu }-h^{*}\right)$. If this output differs from the neuron’s target output, $y^{\mu }$, which is noise-free, the neuron’s input connection weights are updated in four consecutive steps:

(22) $$\begin{array}{l} J_{j}\mapsto J_{j}+\frac{\gamma h}{N}\left(2y^{\mu }-1\right)X_{j}^{*\mu },\;j=1,...,N\\ J_{j}\mapsto J_{j}\theta \left(J_{j}g_{j}\right)\\ J_{j}\mapsto J_{j}+\left(w-\frac{1}{N}\sum_{j=1}^{N}\left|J_{j}\right|\right)g_{j}\\ J_{j}\mapsto J_{j}\theta \left(J_{j}g_{j}\right) \end{array}$$

The first line in Equation 22 is a stochastic perceptron learning step (Rosenblatt, 1962), in which parameter *γ* is referred to as the learning rate. The second line enforces the sign constraints, while the last two lines implement the homeostatic *l*_1_-norm constraint and are equivalent to the soft thresholding used in LASSO regression (Tibshirani, 1996). In contrast to the standard perceptron learning rule, Equation 22 uses noisy inputs and enforce sign and homeostatic constraints at every learning step. They can be used to learn temporally correlated input-output network states, including auto-associations.

By including input spiking errors, synaptic and intrinsic noise in the condition that triggers the learning step outlined in Equation 22, the learning rule implicitly depends on the model parameters $\left\{r_{j}\right\}$, $\left\{\beta_{syn,\;j}\right\}$ describing the fluctuations in the neuron’s inputs (indexed with *j*), and the parameter $\beta_{int}$ which describes the neuron’s intrinsic noise. Because Equation 22 is designed to approximately minimize the neuron’s output spiking error probability for a given memory load (see Materials and Methods), which at capacity matches the desired output error probability of the neuron, *r*, the learning rule also depends implicitly on fluctuations in the neuron’s output.

Figure 6 compares the theoretical solution obtained with the replica method in the *N* → ∞ limit with numerical solutions for networks of *N *=* *200, 400, and 800 neurons obtained with nonlinear optimization and the perceptron-type learning rule. Figure 6*A* shows that the perceptron-type learning rule sometimes fails to find a solution to a feasible learning problem, i.e., a problem that can be solved with nonlinear optimization. Yet, even in such cases, the perceptron connection weights in a steady state (after 10^6^ learning epochs) are well-correlated with the nonlinear optimization weights (Fig. 6*B*). Therefore, though the perceptron-type learning rule is not as efficient as nonlinear optimization, it can find an approximate solution to the learning problem. Consistent with this conclusion, the associative memory storage capacity of a neuron loaded with the perceptron-type learning rule is 15–18% lower than that loaded with nonlinear optimization, and the two methods lead to similar structural and dynamical network properties (Fig. 6*C*, red and blue bars). The scales of non-zero inhibitory and excitatory connection weights according to the replica calculation are primarily determined by *w*, inhibitory/excitatory connection probabilities, and fractions of these inputs (Eq. 14, last line), and this agrees with the results of nonlinear optimization and perceptron learning.

### Properties of heterogeneous associative networks

**Figure 7.**
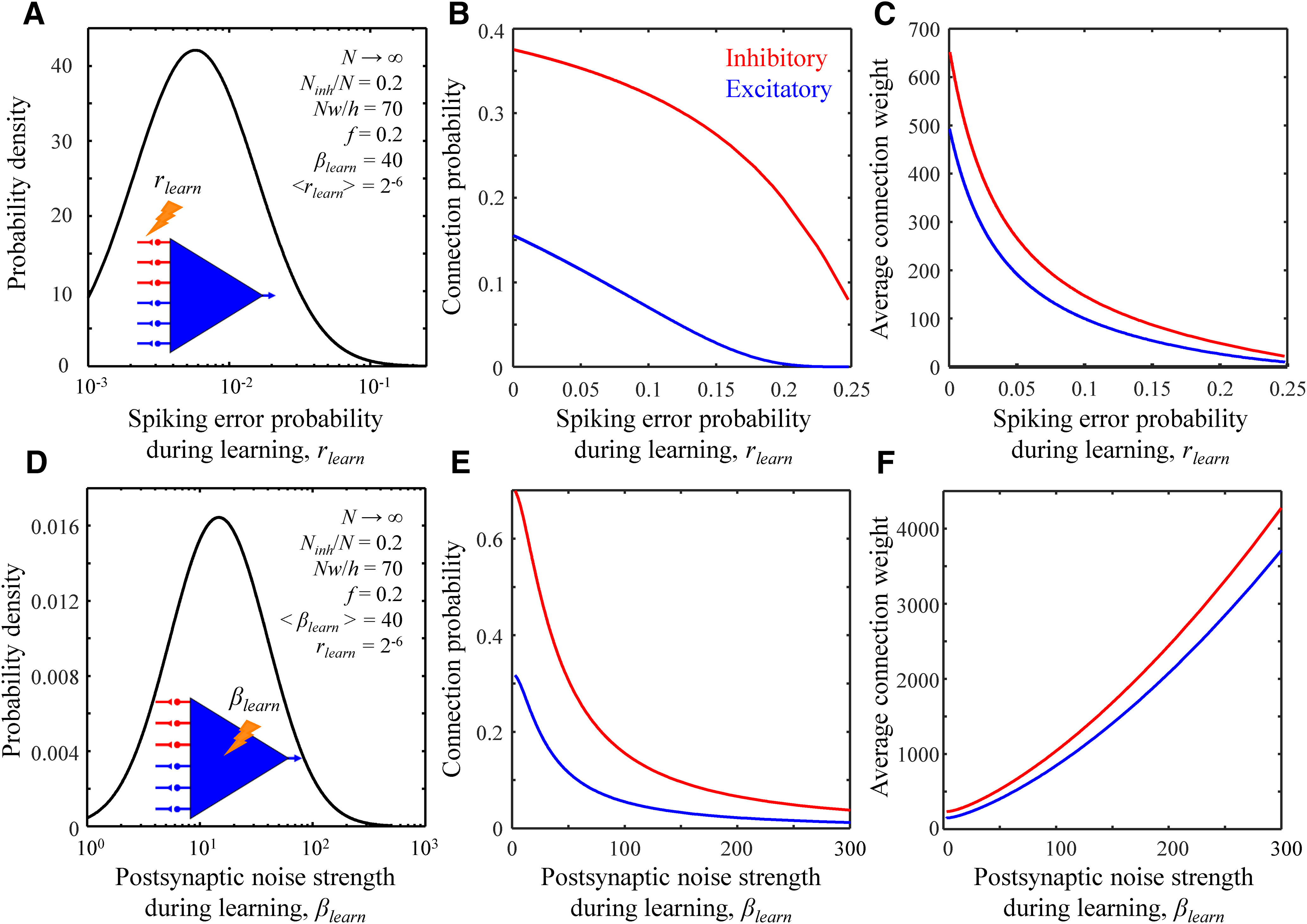
Properties of connections in associative networks of heterogeneous neurons. ***A–C***, Connection probability (***B***) and average non-zero connection weight (***C***) for inhibitory (red) and excitatory (blue) connections in a network of neurons with distributed spiking error probabilities and homogeneous in all other parameters. The spiking error probabilities of inhibitory and excitatory inputs during learning were randomly drawn from the log-normal distribution shown in ***A***. Unreliable inputs have lower probabilities and weights. The parameters of the associative network are shown in ***A***. The values of *β_learn_* and *<r_learn_*> correspond to the green asterisk from Figure 1*F*. ***D–F***, Same for a network of neurons with heterogeneous postsynaptic noise strengths. The postsynaptic noise strengths of neurons during learning were randomly drawn from the log-normal distribution shown in ***D***. Noisier neurons receive stronger but fewer inhibitory and excitatory inputs.

The associative learning model, Equation 1, makes it possible to investigate the properties of networks composed of heterogeneous populations of inhibitory and excitatory neurons. Specifically, we examined the effects of distributed spiking error probabilities and distributed synaptic and intrinsic noise strengths on properties of connectivity at critical capacity. Figure 7*A–C* shows that in networks of neurons with heterogeneous spiking error probabilities (homogeneous in all other parameters), the probabilities and weights of inhibitory and excitatory connections monotonically decrease with increasing *r_learn_*. Therefore, as may have been expected, connections originating from more unreliable neurons (higher *r_learn_*) are more likely to be depressed and/or eliminated during learning. Properties of networks of neurons with distributed synaptic and intrinsic noise strengths (homogeneous otherwise) depend on the combination of these parameters in the form of the postsynaptic noise strengths, *β_learn_*. Figure 7*D–F* show how connection probabilities and average connection weights depend on *β_learn_*. Like in the previous case, connections onto noisier neurons (higher *β_learn_*) are less probable. Here, however, the average inhibitory and excitatory connection weights increase with *β_learn_* because of the homeostatic *l*_1_-norm constraint (Eq. 1).

Motivated by the agreement between the results of the associative learning model and cortical measurements, we put forward two predictions that can be tested in future experiments. First, we predict that in cortical networks, inhibitory and excitatory connections originating from more unreliable neurons or neuron classes must have lower connection probabilities and average uPSPs (Fig. 7*B*,*C*). Second, we predict that connections onto noisier neurons or neuron classes must have lower connection probabilities but higher average uPSPs (Fig. 7*E*,*F*).

## Discussion

We examined a network model of inhibitory and excitatory neurons loaded to capacity with associative memory sequences in the presence of errors and noise. First, we showed that there is a trade-off between the capacity and reliability of stored sequences which is controlled by the levels of errors and noise present during learning. For an optimal trade-off, as judged by the amount of information contained in the retrieved sequences, noise must be present during learning. Second, as synaptic connectivity of neurons changes during learning (Holtmaat and Svoboda, 2009), it is not unreasonable to expect that the requirement of reliable memory retrieval is reflected in the properties of network connectivity and, consequently, the activity of neurons in the brain. Interestingly, local neural networks in the mammalian cortical areas have many common features of connectivity and network activity (Zhang et al., 2019b). We showed that these network properties in the model emerge all at once during reliable memory storage. Third, as levels of errors and noise can differ across individual neurons or neuron classes, we examined the properties of model networks composed of heterogeneous neurons and made two salient predictions regarding the connectivity of neurons operating with relatively high levels of errors and noise.

**Figure 8.**
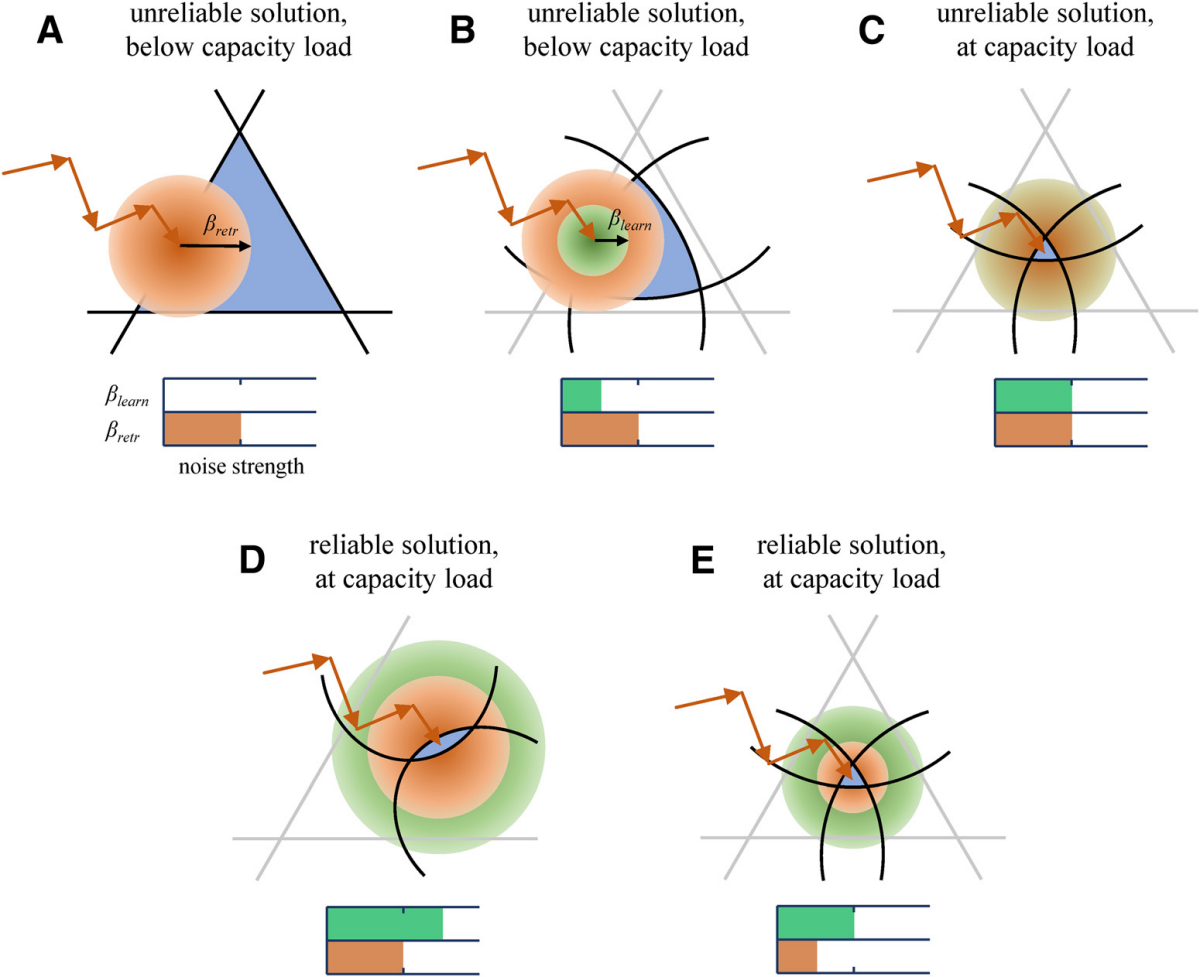
Increasing the noise strength during learning and decreasing it during memory recall lead to more reliable solutions. ***A***, The associative learning problem for a below capacity load in the absence of noise during learning, *β_learn_* = 0. The solution region (blue) is bounded by hyperplanes corresponding to the individual associations (black lines). The learning phase (red arrows) ends as the connection weight vector enters the solution region. The solution shown in ***A*** is unreliable because noise during memory retrieval (red cloud of radius *β_retr_*) can move it outside the solution region with high probability. ***B***, Adding noise during learning (green cloud of radius *β_learn_*) transforms the association hyperplanes (gray lines) into hypersurfaces (black lines; Eq. 1), reducing the solution region and forcing the connection weight vector further away from the hyperplanes. This increases solution reliability. ***C***, The continued increase of the noise strength improves reliability as the solution region shrinks to zero. At this noise strength, the memory load is at capacity. A further increase in reliability can be achieved by increasing the noise strength during learning (***D***) or decreasing it during retrieval (***E***). In the former case, the memory load must be reduced to match the reduction in capacity.

This study incorporates a comprehensive description of errors and noise into the model of associate sequence learning by recurrent networks of neurons with biologically inspired constraints. It shows that errors and noise during learning can be beneficial, as they can increase the reliability of loaded memories to fluctuations during memory retrieval. Because errors and noise are both free and unavoidable harnessing their power, rather than trying to suppress it, may be an efficient way of improving the reliability of memories in the brain. This mechanism is illustrated in Figure 8. When the associative memories are loaded at a below capacity level, the solution region of Equation 1 is comparatively large. A solution, e.g., a vector of connection weights of a neuron obtained with a perceptron-type learning rule, may be located near the solution region boundary. Such a solution is deemed unreliable because a small amount of noise during memory retrieval can move it outside the solution region, resulting in spiking errors that can disrupt the associative sequence retrieval process (Fig. 8*A*). By adding noise during learning, the solution can be forced to move away from the boundary, thus making it more reliable (Fig. 8*B*). However, increasing the noise strength reduces the neuron’s capacity, and at a certain strength, the capacity and memory load are guaranteed to match (Fig. 8*C*). A further increase in noise strength can improve the reliability even more, but at the expense of the memory load as the latter must remain at or below the capacity (Fig. 8*D*). An alternative way of improving reliability is by suppressing noise during memory retrieval (Fig. 8*E*). Incidentally, it has been shown that visual attention that improves behavioral performance reduces the variability in spike counts of individual neurons in Macaque V4 (Cohen and Maunsell, 2009; Mitchell et al., 2009). Though significant, the amount of reduction is relatively small, suggesting that this mechanism has physical limitations. Using noise during learning can enhance the reliability of stored memories beyond what can be accomplished by attending to the memory retrieval process.

The study of associative memory storage by artificial neural networks has a long history dating back to the seminal works of McCulloch and Pitts, Hebb, Rosenblatt, Steinbuch, Cover, Minsky, and Papert (McCulloch and Pitts, 1943; Hebb, 1949; Rosenblatt, 1957; Steinbuch, 1961; Cover, 1965; Minsky and Papert, 1969). Associative models of binary neurons can be generally categorized into learning models, in which memories are loaded into the network over time using activity-dependent learning rules, and memory storage models, which often bypass the learning phase and focus on memory storage capacity and properties of learned networks. Models of the first type often rely on Hebbian-type learning rules in which connection weights are modified based on activities of presynaptic and postsynaptic neurons (Willshaw et al., 1969; Hopfield, 1982; Tsodyks and Feigel'man, 1988; Amit, 1989; Palm, 2013). Although the general idea of Hebbian learning has been corroborated experimentally and characterized as long-term potentiation/long-term depression, recent studies demonstrated that changes in synaptic efficacy can have a complicated dependence on spike timing, spike frequency, and PSP (Sjöström et al., 2001).

Memory storage models make no assumptions as to the details of the learning rules, provided that they are powerful enough to load memories into the network, and analyze network properties as functions of the memory load and network parameters. An advantage of such models is that they often yield closed-form analytically solutions. One of the first models of this type was solved by Cover (Cover, 1965) who used a geometrical argument to show that a simple perceptron with *N* inputs can learn 2*N* unbiased associations. Later, a general framework for the analysis of memory storage capacity was established by Gardner and Derrida (Gardner, 1988; Gardner and Derrida, 1988) who used the replica theory to solve the problem of robust learning of arbitrarily biased associations. Subsequent studies incorporated sources of noise into the associative learning model and examined the effects of learning on neural network properties. In these studies, the basic associative learning model was extended to include biologically inspired elements, such as sign-constrained postsynaptic connections (inhibitory and excitatory; Kohler and Widmaier, 1991; Brunel et al., 2004; Chapeton et al., 2012), homeostatically constrained presynaptic connections (Chapeton et al., 2015), and robustness to noise which is traditionally enforced through a generic robustness parameter *κ* (Gardner, 1988; Gardner and Derrida, 1988). In particular, Brunel et al. (2004) and Brunel (2016) showed that sparse excitatory connectivity and certain two-neuron and three-neuron motifs develop in networks robustly loaded with associations to capacity and that similar results can be obtained in a model which, in place of *κ*, includes Gaussian intrinsic noise and output spiking errors (see their supplementary material). Rubin et al. (2017) considered presynaptic and intrinsic noise and showed that the balance of inhibitory and excitatory currents emerges at capacity. Zhang et al. (2019b) showed that many structural and dynamical properties of local cortical networks emerge in associative networks robustly loaded to capacity.

This article significantly differs from the above-mentioned studies both in terms of the model and results. First, the model introduced in this article provides a more systematic account of errors and noise by combining input and output spiking errors, synaptic and intrinsic noise. Second, the model allows for the possibility of having different levels of errors and noise during learning and memory retrieval. Third, the model makes it possible to analyze networks of neurons with heterogeneous properties. In terms of model results, we first show how errors and noise during learning facilitate reliable memory retrieval and next produce a comprehensive list of results related to network structure and dynamics that are then compared with the data from local cortical networks to validate the model and make predictions. What is more, our results explain the nature of the robustness parameter, *κ*, used in traditional models (Eq. 16) and show explicitly how it is related to errors and noise present during learning.

The model described in this study assumes that individual neurons learn independently from one another and are loaded with memories to capacity. There is no direct support for these assumptions, but they have been shown to lead to structural and dynamical network properties that are consistent with experimental data (Brunel et al., 2004; Clopath et al., 2010; Chapeton et al., 2012; Brunel, 2016; Zhang et al., 2019b). This study corroborates these assumptions by matching a variety of experimental results with a single set of model parameters. The derived perceptron-type rule mediates learning by modifying connection weights based on local activities of presynaptic and postsynaptic neurons in the presence of errors and noise, which is biologically feasible. However, a supervision signal must be fed to every neuron during learning. This is a major drawback of the presented approach and the supervised learning models in general, as the origins of this signal in the brain remain unknown. The problem can be minimized by feeding the supervision signal to a fraction of neurons in the network while letting the remaining neurons learn in an unsupervised manner (Krotov and Hopfield, 2019). Unsupervised learning can be mediated by local spike timing, frequency, and voltage-dependent rules that are biologically more plausible and can explain many experiments describing functional properties of individual neurons (Clopath et al., 2010). However, unsupervised learning rules are not known to produce the host of structural and dynamical properties of local cortical circuits examined in this study. It would be interesting to find out if a recurrent network composed of unsupervised and supervised neurons can satisfy all the requirements of a biologically realistic learning network.

## Synthesis

Reviewing Editor: Gustavo Deco, Universitat Pompeu Fabra Decisions are customarily a result of the Reviewing Editor and the peer reviewers coming together and discussing their recommendations until a consensus is reached. When revisions are invited, a fact-based synthesis statement explaining their decision and outlining what is needed to prepare a revision will be listed below. Note: If this manuscript was transferred from JNeurosci and a decision was made to accept the manuscript without peer review, a brief statement to this effect will instead be what is listed below.

Reviewer 1:

In this study, by using a computational model, authors found that, errors and noise during learning increased the probability of memory recall, and noise is an essence for retrieval of stored information. They also predicted the correlation between receiving connections and neurons’ noise level. Results from this study are interesting and is helpful to the field.

However, I have some concerns as listed below:

1.I’m not sure why authors kept saying that the model in the study is biologically constrained? My understanding is, the McCulloch-Pitts neurons used in the study was more like logistical model, i.e., a simplified model. The biologically realistic model should be at least of Hodgkin-Huxley (HH) conductance-based. To me, this study is more of a theoretical study.

2.In contrast to what authors stated on P.38, several studies found that inhibitory and excitatory currents are indeed not equal in their magnitudes (such as experimental study by Atallah and Scanziani 2009 in Neuron, Salkoff et al., 2015 in J Neurosci, and recent modeling study by Feng et al., 2019 in eNeuro), and inhibitory current is much higher than excitatory current. Interestingly, it seemed like that in this way of current assignment, information can be transmitted more efficiently, and selectivity of the brain region can be enhanced as well (Feng et al., 2019). So, based on what authors found in the current study, any discussion relating to this would be helpful.

3.Authors used the term “noisy neurons or classes", for the prediction that noisy neurons must have lower connection probabilities but higher connection strength. To better help the field, authors need to interpret the “noisy neurons” more clearly, i.e. is there any cut-off between noisy neurons and non-noisy neurons? To what performance extent, a neuron can be deemed as a noisy type?

4.(minor) If parameter was drawn from log-normal distribution, it is better to plot the distribution in log-scale, such as in Figure 7A and D.

Reviewer 2:

Review report on manuscript eN-TNWR-0302-20X, entitled “Noise in neurons and synapses enables reliable associative memory storage in local cortical circuits"

In many ways this is a carefully written and well informed manuscript on its subject. It has some merit in that the noise model is more realistic than in previous similar works. I was not able to check carefully the theoretical formalism, which takes someone skilled in theoretical physics.

However, I have some major concerns with the paper as it stands now, namely:

1. It ignores a large body of published research on neural associative memories, both from a theoretical and memory capacity point of view as well as network simulations.

2. It is built on a supervised error correction learning scheme (Perceptron rule), though neurobiological research puts forward reward modulated correlation based Hebbian learning and LTP/LTD in the brain as the most likely learning rule.

3. It focuses entirely on sequence learning, though earlier work often considered autoassociative as well as heteroassocative (e.g. sequence) learning also produced by much the same synaptic plasticity mechanisms.

4. The focus is on the local network within 100 microns. This is odd considering that one of the authors were among the pioneers to show that a large part of synapses targeting a cortical pyramidal cell originate from much further away, and this is today a common view among associative memory researchers.

These concerns are further detailed in the following.

Regarding 1:

Neural associative memory has been researched theoretically since the 1950’s (e.g. Steinbuch’s Lernmatrix ) and by many following that (Amit, 1989; Palm, 2013; Schwenker, Sommer, & Palm, 1996; Willshaw, Buneman, & Longuet-Higgins, 1969). It was from the start geared towards simple correlation based Hebbian learning, which was later experimentally characterized and described as LTP/LTD. Sequence learning based on Hebbian learning has been demonstrated many times and more recently in a detailed spiking simulation model (Tully, Lindén, Enrik, Hennig, & Lansner, 2016). The connection to these works needs to be made in this article.

Regarding 2:

The neural networks and machine learning community is dominated by supervised error backpropagation learning, which is proven to have very high, sometimes claimed to be “super-human", learning capacity. The Perceptron learning rule is the earliest form of error correction learning and the outset of this manuscript states clearly how noise during learning can improve robustness/reliability of recall since it increases the margin of the decision. So the entire concept is rooted in this terminology. I believe that the results of the paper shows this rather convincingly. But what would be the conclusion if considering a more biologically plausible learning rule?

The attempts to show convincingly that such error-correction learning is neurobiologically plausible. i.e. likely to be used in the brain, are ongoing but has so far convinced few theoretical or experimental brain researchers. The proposed learning rule requires a noise-free Y(n+1). What is the source of such a detailed desired vector in the brain? For instance, a baby learning to reach produces rather random movements which may occasionally be reinforced by an interesting outcome, e.g. touching the object. I fail to see where the noise-free Y(n+1) comes from in such a situation and how a learning rule that uses it can be described as “biologically plausible”. Correlation based learning in different forms, e.g. standard Hebbian learning or Spike-Timing-Dependent-Plasticity (STDP), are generally more acceptable by these communities. Such learning rules are inferior on standard machine learning benchmarks like MNIST but still have an acceptable performance (Krotov & Hopfield, 2019), i.e. 97.5 % on the unseen testset.

The existence and biological plausibility of correlation based Hebbian learning needs to be acknowledged and discussed somewhere in this article to provide a balanced view on the question of learning rules in relation to models of associative memory in the brain.

Regarding 3:

It is too restrictive to consider only sequence learning albeit sequence learning provides a natural X(n) --> Y = X(n+1) pair, which is not so for e.g. unsupervised forms of learning like concept learning. At least it should be explained how the proposed learning rule can be extended to auto-association, which has been important in most other works on neural associative memory.

Regarding 4:

It may be that conduction delays are not part of the model used, but some comment on the model’s applicability and robustness with respect to a more large-scale distributed associative memory system would be desirable.

For me, it is not acceptable to publish this manuscript unless especially the concerns 1 and 2 are treated in an acceptable manner.

Bibliography

Amit, D. (1989). Modeling Brain Function: The world of attractor neural networks. Cambridge: Cambridge University Press.

Palm, G. (2013). Neural associative memories and sparse coding. Neural Networks, 37, 165-171. https://doi.org/10.1016/j.neunet.2012.08.013

Schwenker, F., Sommer, F. T., & Palm, G. (1996). Iterative Retrieval of Sparsely Coded Associative Memory Patterns. Neural Networks, 9, 445-455.

Tully, P. J., Lindén, H., Enrik, Hennig, M. H., & Lansner, A. (2016). Spike-Based Bayesian-Hebbian Learning of Temporal Sequences. PLoS Computational Biology, 12(5), e1004954. https://doi.org/10.1371/journal.pcbi.1004954

Willshaw, D. J., Buneman, O. P., & Longuet-Higgins, H. C. (1969). Non-holographic associative memory. Nature, 222, 960-962.
